# Immunomodulation for stroke-associated pneumonia: a systematic review of mechanistic insight and emerging therapeutic strategies in animal models

**DOI:** 10.3389/fimmu.2026.1856660

**Published:** 2026-07-16

**Authors:** Xiangyun Chen, Yihe Xu, Yonghong Gao, Xinxing Lai, Rufan Xu, Hongrui Zhang, Na Li, Zhenhong Liu, Ying Gao

**Affiliations:** 1 Dongzhimen Hospital, Beijing University of Chinese Medicine, Beijing, China; 2 Institute for Brain Disorders, Beijing University of Chinese Medicine, Beijing, China; 3 College of Traditional Chinese Medicine, Beijing University of Chinese Medicine, Beijing, China; 4 Key Laboratory of Chinese Internal Medicine of Ministry of Education, Dongzhimen Hospital, Beijing University of Chinese Medicine, Beijing, China

**Keywords:** animal model, immunomodulation, immunosuppression, microbial dysbiosis, stroke-associated pneumonia

## Abstract

**Backgrounds and aims:**

Stroke-associated pneumonia (SAP) is a major infectious complication that increases mortality after stroke. Stroke-induced immunosuppression has been proposed as a key driver of SAP, supporting the rationale for immunomodulation. This review systematically outlines the immunopathology of SAP and evaluates immune-targeted strategies.

**Methods:**

Following PRISMA guidelines, we systematically searched PubMed, Scopus, Web of Science, and Embase for studies on the immune mechanisms and immunomodulatory therapies for SAP.

**Results:**

We included 38 studies. Evidence from preclinical models indicates that stroke severity disrupts immune homeostasis, driving SAP through a multi-layered network. Systemic immunosuppression is primarily mediated by sympathetic nervous system (SNS) overactivation, inducing splenic atrophy, lymphocyte apoptosis, and impaired innate immune cells (e.g., iNKT cells), whereas the hypothalamic-pituitary-adrenal (HPA) axis appears to contribute less critically. Furthermore, sustained activation of the cholinergic anti-inflammatory pathway (CAP) may impair pulmonary antimicrobial defense. Active intercellular suppression, as exemplified by monocyte-mediated T cell death, has been identified as a potential mechanism that further compromises immunity. Emerging evidence also implicates disruption of the gut–lung axis (e.g., intestinal barrier dysfunction and microbial dysbiosis), local pulmonary alterations, and dysregulation of key immune molecules (e.g., α-MSH, CD147) as potential contributors to SAP development. Interventions targeting these mechanisms (e.g., iNKT cell activator) have shown promise in preclinical models.

**Conclusion:**

This review systematically synthesizes the mechanistic basis of SAP and identifies emerging immunomodulatory interventions. While preclinical evidence is encouraging, most strategies remain experimental, and their clinical translation requires considerable further validation.

## 1 Introduction

Stroke ranks as the second leading cause of death globally and a primary cause of long-term disability (1). Its incidence is rising with an aging population and is projected to reach 23 million by 2030 (2). A significant factor exacerbating this socio-medical burden is the high risk of post-stroke infections (3). A systematic review and meta-analysis reported that 30% of acute stroke patients develop infections, with pneumonia rates as high as 10%, which are associated with increased mortality (4). Recent investigations have expanded the understanding of post−stroke immune dysregulation at the molecular and cellular levels. These emerging insights, although primarily centered on brain−intrinsic injury, provide essential background for understanding the systemic immune alterations that predispose to infectious complications such as stroke−associated pneumonia (SAP) (5–7).

Despite being a pressing clinical issue, effective prevention and treatment strategies for SAP remain limited, largely due to an insufficient understanding of its pathogenesis. While antibiotics appear to be a viable option for bacterial infections in stroke patients, the rise of drug-resistant bacteria and potential adverse effects on beneficial gut microbiota pose major challenges (8–10). The failure of preventive antibiotic therapy further underscores the limitations of current treatment strategies (11).

Recent studies have gradually revealed that stroke-induced systemic immunosuppression increases susceptibility to bacterial infections and has been strongly linked to the development of SAP (12, 13). The therapeutic potential of immunomodulatory approaches for SAP has been increasingly demonstrated in animal models. As understanding of the immune system’s role in SAP deepens, developing novel immunomodulatory therapies targeting these mechanisms represents a promising direction for future research. This highlights an urgent need for a systematic integration and update of existing evidence in this field.

Therefore, this systematic review aims to provide a comprehensive overview of immune mechanisms and developing immunomodulation therapies for SAP. The specific objectives are to: (1) systematically summarize key cellular and molecular immune mechanisms underlying the development and progression of SAP, and (2) critically discuss current pharmacological strategies targeting the immune system for preventing or treating SAP. By systematically synthesizing the available evidence, this review seeks to provide a conceptual framework and informed rationale for developing innovative strategies against SAP.

## 2 Methods

### 2.1 Search strategy

Four databases, including PubMed, Embase, Scopus, and Web of Science, were searched up to August 30, 2025. The search strategy employed the following keywords: (stroke AND pneumonia) AND (immunity OR immune system OR immunosuppression OR immunomodulation). The detailed search approaches for different databases are provided in the **Supplementary Material**. Studies published in languages other than English were excluded. This review was conducted in accordance with the Preferred Reporting Items for Systematic Reviews and Meta-Analyses (PRISMA) guidelines. The review protocol was not prospectively registered (e.g., in PROSPERO), which the authors acknowledge as a limitation in transparency.

### 2.2 Eligibility criteria

The inclusion criteria were as follows: (1) studies conducted in experimental stroke models that evaluated outcomes related to pulmonary inflammation or lung infection; (2) studies investigating the immune mechanisms (e.g., immune cells, inflammatory cytokines, signaling pathways) underlying pneumonia induced after stroke; (3) studies investigating therapeutic approaches targeting the immune system for SAP. Studies were excluded if they: (1) focused on immune mechanisms or immunotherapies in other diseases (e.g., non-SAP, infections comorbid with other neurological disorders); (2) did not evaluate outcomes related to pulmonary inflammation or lung infection; (3) were not available in full text or had incomplete data; (4) were commentaries, letters, conference abstracts, reviews, editorials, or non-systematic descriptive articles; (5) were published in languages other than English.

### 2.3 Study selection

Two researchers (X.C. and Y.X.) independently screened the titles and abstracts of articles retrieved through the search strategy. Potentially eligible studies then underwent a full-text assessment against the pre-defined criteria to determine final inclusion. Any disagreements were resolved by consensus with a third researcher (Z. L.).

### 2.4 Data extraction

Two researchers (X.C. and Y.X.) independently extracted data from the included studies, and the accuracy of the extracted data was independently verified by another researcher (Z.L.). The following categories of data were systematically extracted: author, year of publication, animal species used, model, primary outcomes, key findings, and intervention methods. Outcome measures included immune- and infection-related indicators (e.g., bacterial clearance, pulmonary inflammation).

### 2.5 Methodological quality assessment

The risk of bias in animal studies was assessed using SYRCLE’s risk of bias tool (14), which evaluates ten entries covering selection bias, performance bias, detection bias, attrition bias, reporting bias and other sources of bias. Each study was rated as having a “high”, “low”, or “uncertain” risk of bias. Two reviewers (X.C. and Y.X.) independently performed the assessments, with any discrepancies resolved by a third reviewer (Z.L.).

## 3 Results

### 3.1 Overview of included studies

**Figure 1:**
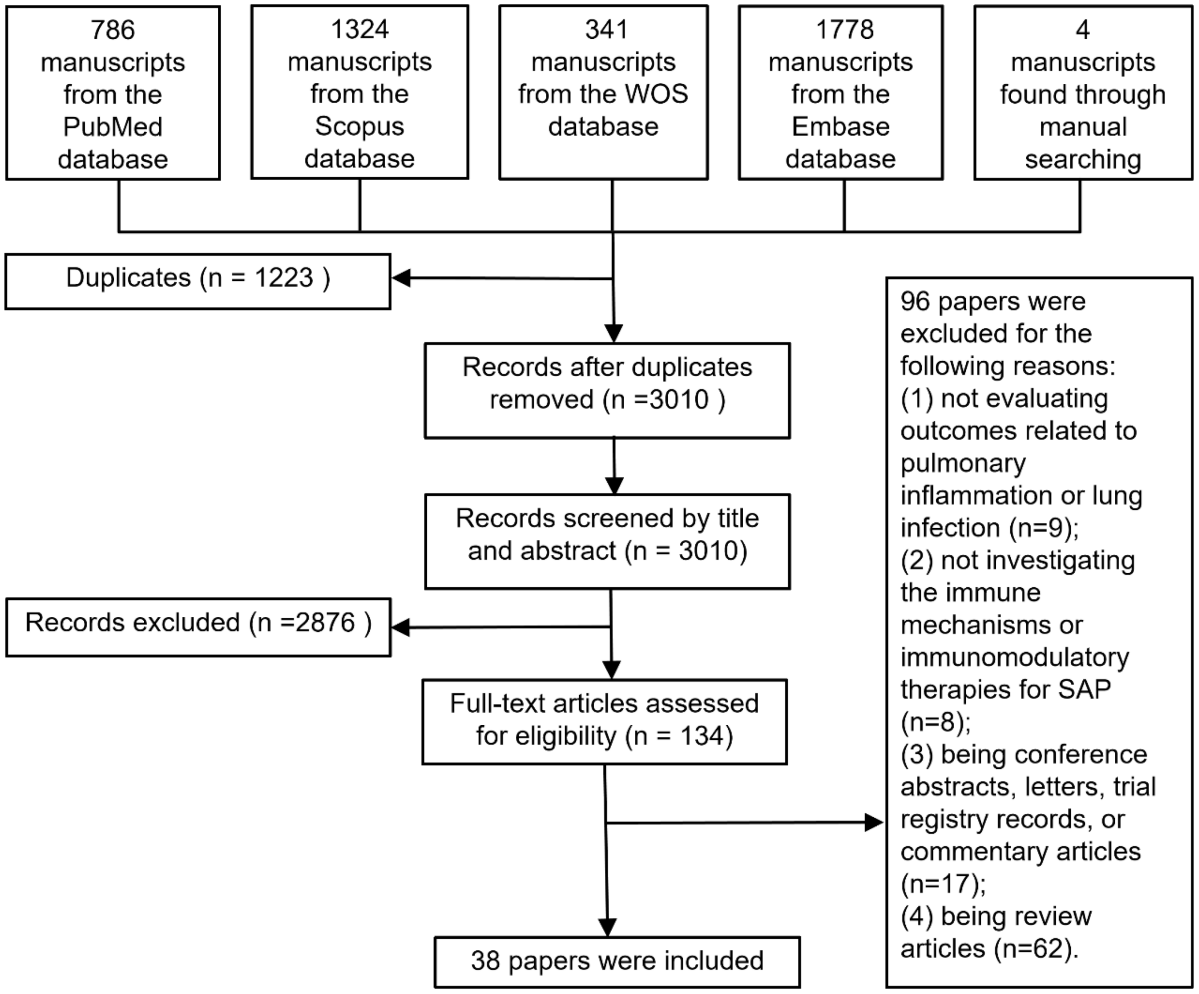
Flow chart of selection process.

**Figure 2:**
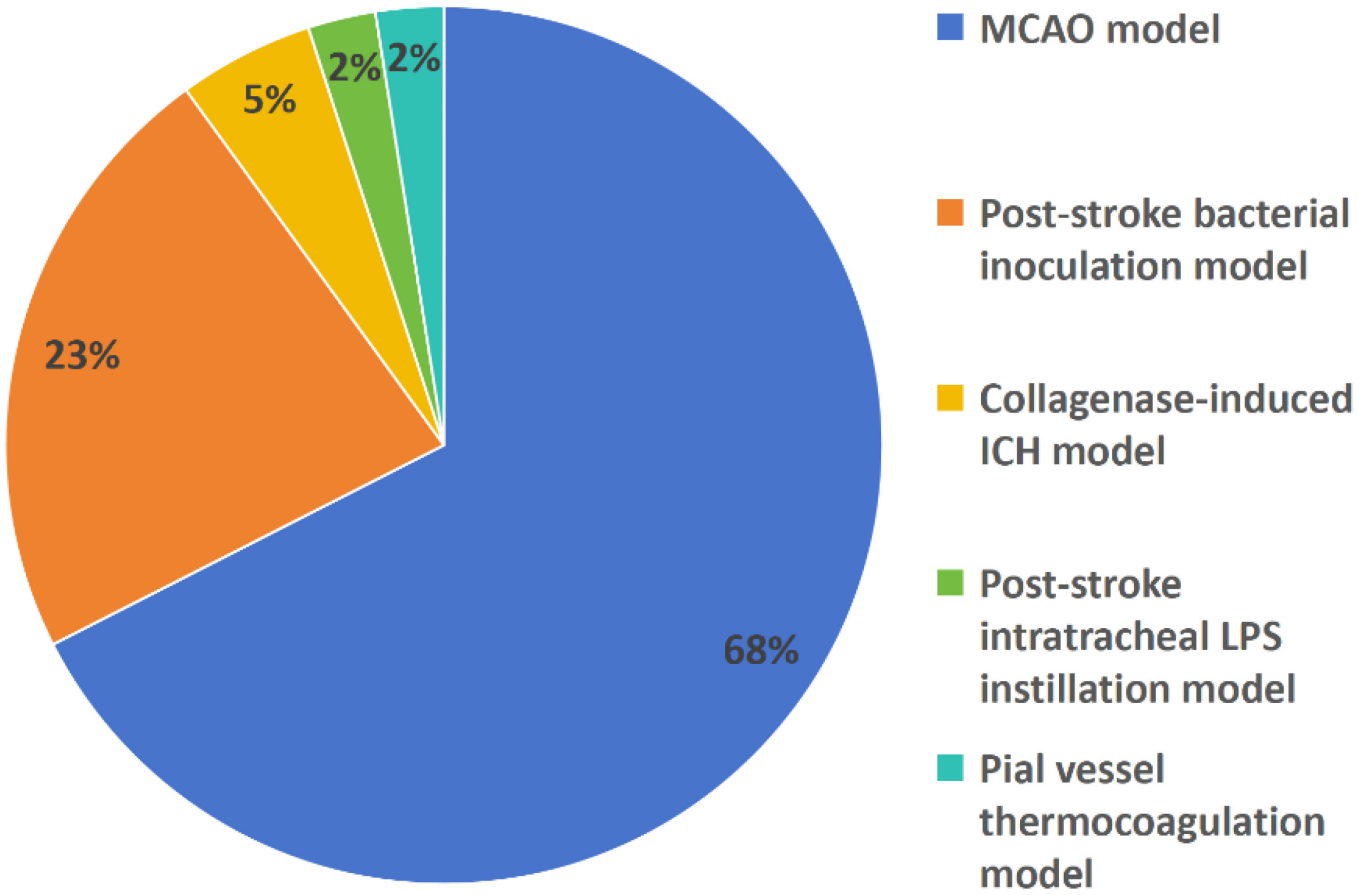
Animal models used in the included studies.

A total of 4233 studies were identified through electronic database searches. After removing duplicates (n = 1223), 3010 studies were screened based on their titles and abstracts. Ultimately, 38 studies were included (**Figure 1**). All 38 included studies were conducted between 2003 and 2025. We summarized the animal models employed in these 38 studies, which primarily include the following five types: the middle cerebral artery occlusion (MCAO) model, post-stroke bacterial inoculation model, collagenase-induced intracerebral hemorrhage (ICH) model, post-stroke intratracheal lipopolysaccharide (LPS) instillation model, and pial vessel thermocoagulation model (**Figure 2**). The most prevalent among these is the focal cerebral ischemia model—MCAO. This model involves inserting a filament via the internal carotid artery to occlude the origin of the MCA, thereby inducing transient or permanent blockage of the vessel. It has been demonstrated to lead to post-stroke immunosuppression and pulmonary infection, the severity of which correlates with the severity of infarction (15–18). Stroke animal models utilizing spontaneous bacterial infection are considered to better mimic the natural occurrence of SAP in clinical settings compared to models involving deliberate bacterial inoculation, thus offering stronger clinical relevance. The second most common model is the composite post-stroke bacterial inoculation model, which has been recently developed for simulating post-stroke pneumonia. This model entails inoculating specific bacteria—such as *Streptococcus pneumoniae*, *Klebsiella pneumoniae*, and *Pseudomonas aeruginosa*, common respiratory pathogens—via intranasal or intratracheal routes following stroke induction. By precisely controlling the bacterial species, inoculum dose, and timing of infection, this model achieves high controllability and reproducibility, making it well-suited for in-depth investigations into the pathogenesis of post-stroke infection, host immune alterations, and related therapeutic strategies. Furthermore, the collagenase-induced ICH model has also been shown to result in secondary pulmonary infection by day 7 post-stroke (19). Additionally, the widespread application of live bacterial pneumonia induction in stroke models is limited due to biosafety concerns and the restricted availability of high-level biosafety laboratories. LPS, a key component of Gram-negative bacterial cell walls, has been extensively used to simulate Gram-negative bacteria-induced acute pulmonary inflammation and injury (20, 21). Consequently, a post-stroke intratracheal LPS instillation model has been developed. This model effectively circumvents the biosafety constraints associated with using live bacteria and can stably and controllably induce pulmonary inflammation characterized by acute neutrophil infiltration and the release of pro-inflammatory cytokines, making it a useful tool for investigating specific immune pathways involved in post-stroke pulmonary inflammation.

### 3.2 Immune mechanisms of SAP

Features of the brain injury itself—particularly infarct volume—have been identified as critical determinants of post-stroke infection risk. Two studies (2009, 2019) focusing on this relationship demonstrate that large infarcts induce more severe systemic immunosuppression, thereby significantly increasing the risk of infectious complications such as pneumonia. The study by Liesz et al. demonstrated the link between infarct volume and immune outcome (22). They found that, compared with mild ischemia, large cerebral infarcts after MCAO led to significant leukopenia, reduced lymphocyte counts in the spleen and other lymphoid organs, and increased splenic lymphocyte apoptosis (22). These mice also exhibited hypothermia and weight loss, ultimately developing pneumonia and sepsis (22). The study further noted that infarct location and laterality had no effect on physiological parameters or immune cell changes, suggesting that infarct volume, rather than location, may be the predominant driver of immunosuppression and infection (22). The work by Shim et al. further substantiated this relationship (18). By adjusting the occlusion duration in the MCAO model to control infarct size, they found that mice with larger infarct volumes had a significantly higher incidence of post-stroke infection, and their pulmonary infections persisted longer. Mechanistically, they observed an inverse correlation between acute-phase infarct volume and circulating leukocyte counts (particularly lymphocytes), and a decreased lymphocyte-to-neutrophil ratio was evident in the lungs of all stroke animals (18). To specifically examine the influence of infarct location, the study also employed a photothrombotic model and, using an innovative systematic analytical approach, concluded that infarct location had no significant impact on infection susceptibility (18). This further supported infarct volume as a predominant factor overriding location, a finding subsequently validated in a clinical setting (18). Taken together, the available evidence suggests that cerebral infarct volume is a key driver of post-stroke infection risk. Large infarcts, by inducing more pronounced systemic lymphopenia and immunosuppression, may markedly increase the incidence and severity of bacterial pneumonia and other complications.

#### 3.2.1 Stroke-induced systemic immunosuppression

##### 3.2.1.1 The sympathetic nervous system

A key mechanism of post-stroke immunosuppression is the excessive activation of the SNS, whose effects are principally mediated by catecholamines—adrenaline, noradrenaline, and dopamine (23). Overall, six included animal studies (2003–2021) have provided evidence that excessive activation of the SNS following stroke plays a central role in mediating systemic immunosuppression and promoting bacterial pulmonary infection.

Early studies identified the important role of the SNS. Prass et al. demonstrated that in MCAO mice, post-stroke catecholamine release triggered lymphocyte apoptosis and impaired interferon-γ (IFN-γ) production, leading to a T helper 1 (Th1)/Th2 imbalance and spontaneous bacterial pneumonia. The β-blocker propranolol alleviated lymphocyte dysfunction and bacterial infection, thereby improving survival in MCAO mice (17). Prass et al. further substantiated the role of the SNS, finding that mice subjected to stroke developed severe pneumonia after inhalation of an extremely low dose of *Streptococcus pneumoniae* (1/1000 of the normal dose), and that this process could be effectively prevented by blocking sympathetic signaling with a β-blocker (24).

Subsequent studies provided deeper insights into specific immune cell targets affected by the SNS. Wong et al. reported that noradrenergic signaling altered the function of hepatic invariant natural killer T (iNKT) cells, thereby mediating systemic immunosuppression. Blocking this neural pathway protected wild-type mice but was ineffective in iNKT cell-deficient mice, whereas activating iNKT cells with α-galactosylceramide reversed immunosuppression and prevented infection (16). Propranolol treatment in MCAO mice also reduced pulmonary neutrophil influx and infection (16). McCulloch et al. discovered that stroke induced a profound loss of splenic B cells, particularly splenic marginal zone B cells, resulting in deficiencies in capturing blood-borne antigens and suppression of circulating IgM. This impaired early antibacterial immunity and led to spontaneous pulmonary infection. Propranolol treatment reversed these impairments and reduced the bacterial burden in MCAO mice (25). Liu et al. showed that splenic NK cells also underwent atrophy and numerical reduction after stroke, a process directly mediated by both the SNS and the hypothalamic-pituitary-adrenal (HPA) axis. In the same study, administration of propranolol and RU486 to block adrenergic and HPA axis innervation of peripheral NK cells significantly enhanced NK cell-mediated immune defense against post-stroke pneumonia (26).

However, the specific adrenergic receptor pathways downstream of the SNS may be complex, and current evidence remains inconclusive. A more recent study by Shim et al. reported that although biochemical markers of SNS activation were elevated post-stroke, under their experimental conditions, neither genetic knockout of the β2-adrenergic receptor (β2AR) nor pharmacological blockade with the non-selective β-blocker propranolol or the selective β2AR antagonist ICI-118551 reduced post-stroke pulmonary infection in wild-type mice. The authors proposed that compensatory mechanisms common in knockout animals, or other pathways acting independently of or in concert with β2AR signaling, might contribute to infection development (15). Given that these findings contrast with the protective effects of propranolol reported in earlier studies, it is possible that the role of β2AR in SAP is model- or context-dependent, and further investigation is required to reconcile these discrepancies.

Collectively, these studies suggest that excessive SNS activation acts as a central driver of post−stroke immunosuppression, although the specific downstream adrenergic pathways and the precise immune cell targets remain to be fully clarified. Despite these unresolved questions, interventions targeting the SNS or its downstream immune effects continue to represent a promising direction for SAP prevention, pending further validation in clinically relevant models.

##### 3.2.1.2 The HPA axis

In the neuroendocrine response to stroke, the perception of inflammatory markers by the hypothalamus is thought to trigger HPA axis activation. This prompts the adrenal zona fasciculata to oversecrete glucocorticoids, which induce lymphocytopenia and shift the balance of inflammatory mediators (27, 28). While glucocorticoids exert anti-inflammatory effects at physiological levels, their excessive elevation compromises host defense, which may explain the association between preadmission systemic glucocorticoid use and an increased risk of short-term mortality after stroke (29).

Two studies have specifically addressed the role of the HPA axis in SAP. Available evidence indicates that the HPA axis is involved in post-stroke immunosuppression, though its role in driving pulmonary infection appears to be less critical than that of the SNS. Prass et al. demonstrated that in MCAO mice, inhibition of the HPA axis via the glucocorticoid receptor blocker RU486 prevented lymphocyte apoptosis. However, in contrast to propranolol treatment, it did not reduce bacterial infection. These results indicate that the role of the HPA axis in SAP may be less critical than that of the SNS (17). The study by Liu et al. indicated that activation of the HPA axis, together with that of the SNS, contributes to post-stroke splenic atrophy and the numerical reduction of peripheral NK cells. Combined administration of RU486 and propranolol to block signaling from both the HPA axis and the SNS significantly enhanced NK cell-mediated immune defense against post-stroke pneumonia, further supporting a role of the HPA axis in the development of SAP (26). Taken together, the HPA axis appears to be a contributory component, rather than the primary driver, of the neuroimmune dysregulation that promotes SAP.

##### 3.2.1.3 The cholinergic anti-inflammatory pathway

The CAP has been proposed as another important mechanism contributing to post-stroke immunosuppression. This pathway is mediated by acetylcholine (ACh) released from the vagus nerve, which binds to the α7 nicotinic acetylcholine receptor (α7nAChR) on macrophages and other immune cells, thereby suppressing the production of pro-inflammatory cytokines (30). However, sustained activation of this pathway following stroke may paradoxically impair essential antimicrobial defenses in the lung tissue while inhibiting excessive inflammation, consequently increasing the risk of infection.

Evidence supporting this dual role was provided by Lafargue et al. In a mouse model of stroke combined with *Pseudomonas aeruginosa* pneumonia, pharmacological inhibition or genetic knockout of α7nAChR attenuated *Pseudomonas aeruginosa*-induced lung injury and mortality, whereas activation of the receptor exacerbated post-stroke lung damage (31). Engel et al. further substantiated the involvement of this pathway by demonstrating that inhibition of cholinergic signaling—either through vagotomy or by using α7nAChR-deficient mice—reversed post-stroke pulmonary immune hypo-responsiveness and prevented the development of pneumonia. These findings indicate that vagus nerve stimulation, via activation of the CAP, compromises antibacterial immunity in the lungs (32).

While α7nAChR appears to be a key mediator, it may not fully account for the complexity of the cholinergic immunosuppressive mechanisms affecting lung defense. Jagdmann et al. reported that individual genetic deletion of the α2, α5, α7, or α9/10 nAChR subunits failed to enhance the clearance of *Streptococcus pneumoniae* in stroke mice (33). This finding suggests that the specific nAChR subunits responsible for impaired pulmonary defense remain to be conclusively identified, and that the involvement of the CAP in SAP may be more nuanced than a single-receptor model would predict. At present, the evidence regarding the precise receptor pharmacology of this pathway remains model-dependent and warrants further systematic investigation.

Collectively, these findings suggest that the CAP functions as an important modulator of post−stroke immune function with a dual role that must be carefully considered in therapeutic development. However, current evidence regarding the precise receptor pharmacology and the conditions under which CAP modulation is beneficial versus harmful remains incomplete. Further systematic investigation is warranted before CAP−targeted strategies can be rationally designed for SAP.

##### 3.2.1.4 Active intercellular immunosuppression

Systemic immunosuppression following stroke arises not only from passive exhaustion processes such as lymphocyte apoptosis, but may also involve active inhibitory interactions between immune cells.

Recent research has identified a potential pathway in this regard. Roth et al. reported a monocyte–T cell interaction that drives bystander T cell death after ischemic stroke. Specifically, stroke was found to induce a Fas ligand (FasL)-expressing monocyte population, which in turn promoted extrinsic T cell apoptosis. This phenomenon was shown to be triggered by absent in melanoma 2 (AIM2) inflammasome-dependent interleukin-1β (IL-1β) secretion following the sensing of cell-free DNA. Pharmacological inhibition of this pathway improved T cell survival and reduced post-stroke bacterial infection. Thus, this study identifies inflammasome-dependent monocyte activation as a previously unrecognized mechanism of post-injury T cell death that challenges the prevailing paradigm regarding lymphocytopenia after injury (34). These findings identify monocyte−mediated T cell apoptosis as a previously unrecognized mechanism contributing to post−stroke lymphocytopenia. While this represents a promising therapeutic target, the evidence is currently derived from a single study and requires independent replication before its broader significance within the immunosuppressive network can be definitively established.

#### 3.2.2 Pulmonary immunological changes

Immunological dysfunction following stroke manifests not only as systemic immunosuppression but also leads to significant alterations in the pulmonary immune microenvironment. Collectively, seven animal studies (2018–2025) indicate that ischemic stroke can induce local pulmonary inflammatory responses and disrupt immune homeostasis, thereby increasing susceptibility to pneumonia.

Pulmonary inflammation can be observed early after stroke. Samary et al. reported that focal cerebral ischemia within 24 hours triggered diffuse alveolar damage, ultrastructural disruption, elevated pulmonary pro-inflammatory mediators (TNF-α, IL-6), and impaired phagocytic function of alveolar macrophages. Notably, serum from stroked animals was sufficient to induce inflammatory cytokine production and reduce phagocytic capacity in naïve macrophages *in vitro*, suggesting that circulating inflammatory mediators may be key contributors to early pulmonary immune dysfunction (35). Austin et al. similarly observed increased macrophages and neutrophils in bronchoalveolar lavage fluid (BALF), along with up-regulated IL-1β mRNA expression in MCAO mice, although no significant histological lung injury was evident (36). Additionally, Xu et al. showed that NOD-like receptor family pyrin domain-containing protein 3 (NLRP3) inflammasome knockout not only alleviated cerebral ischemia-reperfusion injury but also mitigated stroke-associated lung injury by down-regulating pulmonary caspase-1 and IL-1β expression, reducing macrophage/neutrophil infiltration, and suppressing nuclear factor-κB (NF-κB) pathway activation (37). Somogyi et al. provided further evidence that stroke induces pulmonary edema and elevates IL-1β and TNF-α levels in lung tissue (38). Recent studies have further elucidated precise brain–lung regulatory axes. Chen et al. demonstrated that ischemic stroke induces apoptosis of glutamatergic neurons in the hypothalamic paraventricular nucleus (PVN), which attenuates excitatory drive to cholinergic neurons in the dorsal motor nucleus of the vagus (DMV), resulting in reduced pulmonary parasympathetic tone. This impairs the α7nAChR-dependent CAP, thereby releasing inhibition on high mobility group box-1 protein (HMGB1) and ultimately driving excessive inflammation and pneumonia via Toll-like receptor 4 (TLR4)/receptor for advanced glycation end products (RAGE) signaling. Chemogenetic activation of the “DMV–lung” circuit improved lung inflammation, providing causal evidence for this pathway (39).

Stroke also markedly alters the composition and function of pulmonary immune cells. Farris et al. found that after stroke, the proportions of alveolar macrophages, neutrophils, and CD11b+ dendritic cells increased, whereas the proportions of CD4+ T cells, CD8+ T cells, B cells, NK cells, and eosinophils decreased. These shifts were concomitant with reduced levels of multiple pulmonary chemokines, including CCL3, CCL4, CCL5, CCL17, CCL20, CCL22, CXCL5, CXCL9, and CXCL10 (40). Functionally, Wen et al. revealed that stroke leads to pulmonary vascular neutrophil retention and impaired phagocytic capacity, a defect that worsens with aging (41).

In summary, converging evidence from multiple independent studies indicates that stroke can profoundly reshape the local pulmonary immune environment through mechanisms including early release of pro-inflammatory mediators and tissue injury, impaired function of key immune cells (e.g., macrophages and neutrophils), imbalance of immune cell subsets, and dysregulation of specific neuro-immune and inflammasome pathways. These alterations are increasingly recognized as key contributors to the local immunological susceptibility underlying post-stroke pneumonia and highlight potential therapeutic directions aimed at restoring pulmonary immune cell function or modulating pertinent neuro-immune axes.

#### 3.2.3 Gut–lung axis

Immunological dysfunction following ischemic stroke is not confined to the central nervous system and lungs. Emerging evidence suggests that the ‘gut–lung axis’ has been proposed to play a role as an inter-organ communication pathway that may contribute to post-stroke immune dysregulation, although its precise role remains to be fully defined. Collectively, three studies (2016–2023) indicate that stroke can induce intestinal barrier dysfunction and gut microbiota dysbiosis, which may in turn influence systemic and pulmonary immune status through alterations in microbial composition and metabolites.

Early studies provided evidence for the existence of a gut–lung axis. Stanley et al. showed in a mouse model of ischemic stroke that bacteria detected in the lungs originated from the host intestine. Stroke-induced increases in intestinal permeability and functional impairment that occurred before bacterial dissemination to peripheral tissues, suggesting that stroke may promote the selective translocation and spread of specific bacterial strains from the gut microbiota (42). Subsequently, Zhang et al. described the dynamic timeline of this axis in a model of ICH: systemic immunosuppression peaked on day 3 after stroke, followed by progressive intestinal barrier disruption, which was associated with gut-derived bacterial translocation to the lungs and infection by day 7, suggesting a potential critical window for early intervention (19).

However, emerging research offers a more nuanced perspective. Diaz-Marugan et al. found that stroke induced lymphocytopenia and widespread colonization of the lungs and other organs by opportunistic commensal bacteria. This effect was associated with a compromised intestinal epithelial barrier, accompanied by local activation of complement and NF-κB, a reduction in intestinal regulatory T cells, and a shift in gut lymphocytes toward γδ T-cell and Th1/Th17 phenotypes. Furthermore, stroke increased conjugated bile acids (BAs) in the liver but decreased levels of both BAs and short-chain fatty acids (SCFAs) in the gut—BAs and SCFAs are signaling metabolites derived from the gut microbiota that mediate interorgan communication. The intestinal microbiota composition shifted, marked by a decrease in fermenting anaerobic bacteria and an expansion of opportunistic facultative anaerobes, particularly *Enterobacteriaceae*. Notably, while anti-inflammatory treatment with an NF-κB inhibitor completely abolished this stroke-induced bloom of *Enterobacteriaceae* in the gut microbiota, it did not prevent their colonization in the lungs post-stroke. These findings suggest that stroke disrupts the neuro-immune-metabolic network that maintains homeostasis, thereby promoting the expansion of opportunistic commensals in the gut. However, this intestinal bacterial expansion itself does not directly lead to infection after stroke (43).

In summary, initial evidence suggests that the gut–lung axis may participate in post-stroke immune regulation, with studies demonstrating that stroke disrupts the intestinal barrier and may promote bacterial translocation. Nevertheless, the causal relationship between gut microbial alterations and lung infection remains unresolved, and the causal contribution of gut-derived changes to pulmonary infection remains an active area of investigation requiring further elucidation. Some studies indicate a direct contribution of bacterial translocation to pulmonary infection, while others show that abolishing intestinal dysbiosis does not prevent lung colonization. These contradictory findings may be explained by differences in experimental models, stroke severity, and the specific microbial strains investigated, and they underscore the need for further elucidation before definitive conclusions can be drawn.

#### 3.2.4 Immune molecules and signaling pathways

This section synthesizes findings from four animal studies (2008–2021) suggesting that susceptibility to post-stroke pneumonia may be regulated by specific immune molecules and signaling pathways.

In an initial study, Schulte-Herbrüggen et al. found that the neuropeptide alpha-melanocyte-stimulating hormone (α-MSH), which is rapidly up-regulated after cerebral ischemia, was associated with increased susceptibility to spontaneous pneumonia in mice via the α-MSH receptor-1 (MC-1R). Antagonizing MC-1R reduced pulmonary bacterial burden, whereas exogenous α-MSH supplementation exacerbated infection. Notably, the α-MSH-mediated suppression of antibacterial defense was independent of its modulatory effects on pulmonary cellular immune responses (e.g., elevated IL-10, decreased IFN-γ), suggesting the involvement of additional mechanisms (44). Ritzel et al. focused on the immunoinhibitory cluster of differentiation 200–CD200 receptor 1 (CD200–CD200R1) signaling axis. Loss of this signaling pathway markedly increased the incidence of spontaneous respiratory bacterial infections after stroke and impaired timely resolution of neuroinflammation, identifying this pathway as an endogenous mechanism important for maintaining immune homeostasis and defending against SAP (45). Similarly, Jin et al. provided preliminary evidence identifying CD147 as a potential regulator of pulmonary immune dysregulation after stroke. Inhibition of CD147 ameliorated aberrant pulmonary inflammation and immune responses and reduced post-stroke bacterial infection (46). Furthermore, Roth et al. proposed that post-injury immunosuppression and secondary infection may be driven, at least in part, by an AIM2 inflammasome-dependent signaling cascade. AIM2 inflammasome activation led to the release of circulating IL-1β, up-regulation of FasL expression on monocytes, and initiation of Fas-dependent T-cell apoptosis. Inhibition of this pathway was associated with reduced bacterial infection and increased T-cell survival after experimental stroke (34). These emerging pathways represent promising targets, though their clinical significance remains to be validated through further investigation.

**Figure 3:**
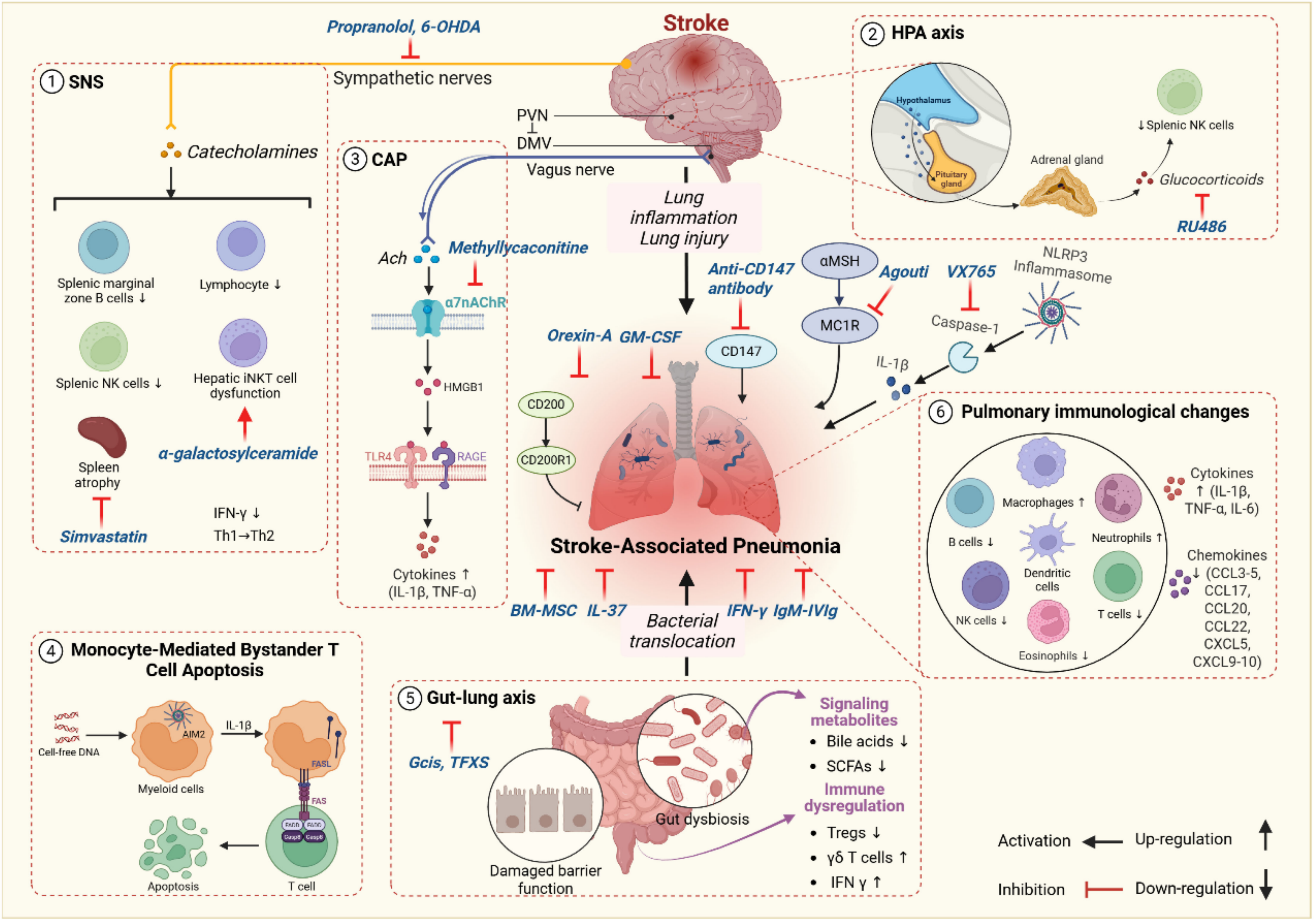
Immune mechanisms of SAP. This schematic, based on experimental animal studies, systematically summarizes the key immune mechanisms proposed to underlie SAP. Stroke onset, particularly when severe, is thought to act as a central driver that disrupts systemic and local immune homeostasis. Systemic immunosuppression has been linked to neuroendocrine activation, including excessive SNS output and HPA axis signaling. This may lead to splenic atrophy, apoptosis of lymphocytes (T, B, and NK cells), and functional impairment of innate immune cells (e.g., iNKT cells). Concurrently, the CAP, while exerting anti-inflammatory effects, has been proposed to paradoxically suppress pulmonary antimicrobial defenses when overactivated. An active immunosuppressive mechanism identified in recent studies involves stroke-induced, AIM2 inflammasome-dependent monocyte activation, which drives FasL-mediated bystander T-cell apoptosis. Parallel to these systemic changes, emerging evidence suggests that stroke may disrupt the gut–lung axis, potentially contributing to intestinal barrier dysfunction, microbial dysbiosis, and translocation of opportunistic bacteria. Locally, stroke has been shown to induce pulmonary inflammation, alter immune cell composition (e.g., increased neutrophils/alveolar macrophages, decreased lymphocytes), and impair phagocytic function. Specific neuro-immune circuits (e.g., the hypothalamic PVN–DMV–lung pathway) and immune-modulatory molecules (e.g., α-MSH, CD200–CD200R1, CD147) have been implicated in the development of SAP. Together, these multi-level disturbances—spanning neurogenic, systemic, intestinal, and pulmonary compartments—are proposed to synergistically compromise host defense, ultimately predisposing to SAP. Potential immunomodulatory targets are also indicated in the figure, providing directions for developing novel therapeutic strategies. (Created in BioRender. Liu, Z. (2026) https://BioRender.com/yog2fxh).

In summary, these initial studies suggest that susceptibility to post-stroke pneumonia may involve a complex molecular network encompassing neuropeptides (e.g., α-MSH), immune checkpoints (e.g., CD200–CD200R1), and inflammasome signaling (e.g., AIM2). Based on current, albeit limited, evidence, these pathways may collectively contribute to shaping the immunosuppressive microenvironment at multiple levels. Further independent validation is required to substantiate their roles and establish their therapeutic relevance. A schematic overview of the key immune mechanisms implicated in SAP is presented in **Figure 3**. A detailed summary of the immune mechanisms identified in animal studies is provided in **Table 1**.

**Table 1:** The immune mechanisms of SAP in animal studies.

Mechanism classification	Author (year)	Animal	Stroke model	SAP model	Primary outcomes	Key findings
Activation of SNS	Prass et al. (2003) (17)	Gender-mixed 7-week-old or older SV129/J mice or C57BL/6J mice weighing 18–22 g; B6.129P2-Tcrb^tm1Mom^ (αβ T cell–deficient), B6.129P2-Tcrd^tm1Mom^ (γδ T cell–deficient), B6.129S7-INFγ^tm1Ts^ (IFN-γ–deficient).	tMCAO for 60 min	Spontaneous pneumonia (3 d after ischemia)	Apoptotic loss of lymphocytes↑; IFN-γ↓; Bacterial infections↑; Mortality↑.	A catecholamine-mediated defect in early lymphocyte activation is the key factor in the impaired antibacterial immune response after stroke.
	Prass et al. (2006) (24)	129S6SvEv mice (aged 9–13 weeks, mixed gender)	tMCAO for 60 min	Aspiration pneumonia (intranasal application of 20 microL of a defined suspension of Streptococcus pneumoniae in PBS 4 or 14 days after MCAO)	Experimental stroke propagates bacterial aspiration from harmless intranasal colonization to harmful pneumonia.	Immunodepression by sympathetic hyperactivity is essential for progression of bacterial aspiration to pneumonia.
	Wong et al. (2011) (16)	Balb/c and CD1d-deficient (Cd1d^-/-^, Balb/c background) mice; Cxcr6^gfp/+^ mice; male mice of 8–12 weeks old.	tMCAO for 30 min	Spontaneous pneumonia (24 hours after MCAO)	Behavioral changes in hepatic iNKT cells; A general switch in systemic immunity from Th1-to Th2-type in the early stages of reperfusion after stroke; Neutrophilic infiltration into lungs↑; Mortality↑	Stroke mediates systemic immunosuppression by altering the function of liver iNKT cells through noradrenergic neurotransmitters.
	McCulloch et al. (2017) (25)	Male 8–10-week-old C57BL/6 J mice; Ccr2^RFP/+^ transgenic mice	tMCAO for 40 min	Spontaneous bacterial lung infection	Splenic marginal zone B cells↓; Capturing blood-borne antigen↓; circulating IgM↓; spontaneous bacterial lung infection↑.	Adrenergic-mediated loss of Splenic marginal zone B cells contributes to the infection-prone state after stroke.
	Liu et al. (2017) (26)	Adult male 10-to 12-week-old mice Male C57BL/6; Rag2^-/-^Il2rg^-/-^; nAChR β2-subunit genetically ablated (Chrnb2^-/-^) mice; Cd1d^-/-^NK1.1-tdTomato mice	tMCAO for 60 min	Spontaneous pneumonia	Spleen volumes↓(24 hour after brain ischemia); Resolution of splenic atrophy (day 7 after brain ischemia); Lymphopenia occurred in mice subjected to MCAO and subsequently recovered to normal lymphocyte counts at day 7 after MCAO; In the periphery, NK cell numbers decreased rapidly after brain ischemia, and then returned to basal numbers; In the brain, highest number of NK cells appeared during early stages of ischemia, then contracted afterward.	The activation of catecholaminergic leads to splenic atrophy and contraction of NK cell numbers in the periphery through a modulated expression of SOCS3.
	Shim et al. (2021) (15)	Adult male C57Bl/6J mice of 7–12 weeks; β2AR deficient (*Adrb2*^-/-^) mice	Permanent MCAO	Spontaneous pneumonia	Plasma catecholamines↑; UCP-1 activity↑; Lung infection↑.	Other mechanisms independent or in combination with β2AR activation contribute to the development of post-stroke lung infection.
Activation of HPA	Liu et al. (2017) (26)	Adult male 10-to 12-week-old mice Male C57BL/6;Rag2^-/-^Il2rg^-/-^; nAChR b2-subunit genetically ablated (Chrnb2^-/-^) mice; Cd1d^-/-^NK1.1-tdTomato mice	tMCAO for 60 min	Spontaneous pneumonia	Splenic atrophy↑; NK cell numbers in the periphery↓; NK cell-mediated immune defense against post-ischemic pneumonia↓.	Brain ischemia weakens the immune defense mediated by NK cells against ischemic pneumonia by activating the HPA axis.
Activation of CAP	Lafargue et al. (2012) (31)	C57BL/6 mice; Male mice aged 10-to 14-week-old; α7-knockout mice.	tMCAO for 60 min	Followed by 24 h of reperfusion, then 50 μL of PBS containing 1×10^7^ CFU of *Pseudomonas aeruginosa* was instilled into both lungs through the trachea via the mouth.	*Pseudomonas aeruginosa*-induced lung injury and mortality↑.	Activation of the α7nAChR plays an important role in mediating the systemic immunosuppression observed after stroke and directly contributes to more severe lung damage induced by *Pseudomonas aeruginosa*.
	Engel et al. (2015) (32)	Male C57BL6/J mice; sex-mixed α7nAChR knockout mice; wild-type littermates	tMCAO for 60 min	Spontaneous pneumonia	Pulmonary immune responsiveness↓; Pneumonia after stroke↑;	Activation of Cholinergic pathways inhibit the post-stroke pulmonary antibacterial response by acting on α7nAChR.
	Jagdmann et al. (2020) (33)	Male α2 nAChR KO; α5 nAChR KO; α7 nAChR KO; α9/10 nAChR KO; C57BL/6J mice.	tMCAO for 60 min	Bronchoscopy-Guided application of *S. Pneumoniae* 3 days after MCAO	Deficiency of various nAChRs does not contribute to an enhanced clearance of a Gram-positive pathogen causing post-stroke pneumonia in mice.	A single nAChR is not sufficient to mediate the impaired pulmonary defense against S. pneumoniae after experimental stroke.
Active intercellular immunosuppression	Roth et al. (2021) (34)	C57BL6/J mice; Aim2^-/-^ mice; Rag-1^-/-^ mice; Casp1^-/-^ mice The ASC-Citrine reporter mice; Pycard^-/-^ mice; Myeloid-specific ASC-deficient mice; Cx3Cr1^GFP/+^ mice Fas^lpr^ mice.	tMCAO for 60 min	The inoculum (10^6^ CFU of *S. pneumoniae* or 2x10^5^ CFU of *K.pneumoniae*) was administered with a pipette onto the nostrils of the mice.	T cell survival↓; Post-stroke bacterial infections↑.	AIM2 Inflammasome-dependent monocyte activation as a previously unstudied cause of T cell death after injury and challenges the current paradigms of post-injury lymphopenia.
Pulmonary immunological changes	Samary et al. (2018) (35)	male Wistar rats (weight 350–400 g)	Thermocoagulation of pial vessels over the right primary sensorimotor cortex	/	The phagocytic ability of macrophages↓; gene expression and protein levels of TNF-α and IL-6 in brain and lung tissue homogenates↑; protein levels of TNF-α in BALF and plasma↑; IL-6 in plasma↑; Gene expression of IL-6 in macrophages and endothelial cells↑;	Focal ischemic stroke is associated with brain-lung crosstalk, leading to increased pulmonary damage and inflammation, as well as reduced alveolar macrophage phagocytic capability, which seems to be promoted by systemic inflammation.
	Austin et al. (2019) (36)	Male 7–12-week-old C57BL/6 mice	MCAO for either 50 min or 60 min	/	Macrophages and neutrophils in BALF↑; IL-1β mRNA in the lung tissue↑;	Focal ischemic stroke causes lung inflammation, but not ALI.
	Farris et al. (2019) (40)	8 to 12-week-old male C57BL/6J mice, weighing 25 to 30 g	tMCAO for 60 min	Spontaneous bacterial infection and tissue damage following tMCAO were not observed.	The percentage of alveolar macrophages, neutrophils, and CD11b+ dendritic cells in the lungs↑; The percentage of CD4+ T cells, CD8+ T cells, B cells, NK cells, and eosinophils in the lungs↓; Multiple chemokines in the lungs, including CCL3, CCL4, CCL5, CCL17, CCL20, CCL22, CXCL5, CXCL9, and CXCL10↓;	Ischemic stroke directly impacts pulmonary immunity.
	Wen et al. (2022) (41)	Young (8–10 week of age) and advanced age or “older” (12–15 month of age) male C57BL/6J mice.	tMCAO for 20 min	Spontaneous bacterial infection	Older (12–15 mo of age) mice had elevated lung bacterial infection and inflammatory damage after stroke when compared with young (8–10 wk of age) counterparts; in younger mice, stroke promoted neutrophil arrest in pulmonary microvessels, but this response was not seen in older poststroke mice; bacterial phagocytosis by neutrophils in the lung microvasculature was reduced by both aging and stroke; stroke alone did not negatively impact neutrophil migration, but that the combination of increased age and stroke led to reduced effectiveness of neutrophil chemotaxis.	Stroke-induced impairment in pulmonary intravascular neutrophil function, a response exacerbated with aging.
	Xu et al. (2022)a (37)	Wild-type C57BL/6J mice (aged 8–10 weeks, 22–25 g; NLRP3-KO mice.	tMCAO for 60 min	/	Cerebral ischemia-reperfusion injury↑; Lung inflammation↑; Caspase-1 and IL-1β expression↑; Macrophage and neutrophil infiltration in the lung↑; Phosphorylated p65↑;	NLRP3 promotes lung injury caused by cerebral ischemia-reperfusion.
	Somogyi et al. (2024) (38)	Male SD rats; 8–10 weeks (286–388 g)	Permanent MCAO; t MCAO	/	IL-1β and TNF-α in the lung tissue ↑;	Deteriorations in the respiratory tissue mechanics develop after permanent focal ischemia.
	Chen et al. (2025) (39)	Male SD rats; weighing 200–300 g	Permanent MCAO	/	Inflammatory cell infiltration in the alveoli and pulmonary interstitium, including macrophages and neutrophils↑; TNF-α, IL-1β↑; HMGB1 ↑;	Acute ischemic stroke induces the apoptosis of DMV^ACh^-innervating PVN^CaMKII^ neurons, and subsequently promotes the immune cells infiltration and inflammatory cytokines increases by acting on the α7nAChR-HMGB1 pathway in the pulmonary ganglion neurons, which leads to pulmonary dysfunction.
Gut-lung axis	Stanley et al. (2016) (42)	7-to-10-week-old male C57BL/6J mice	tMCAO for 60 min	Spontaneous lung bacterial infection	The source of the bacteria forming the microbial community in the lungs of post-stroke mice was indeed the host small intestine; stroke-induced gut barrier permeability and dysfunction preceded the dissemination of orally inoculated bacteria to peripheral tissues.	Stroke promotes the translocation and dissemination of selective strains of bacteria that originated from the host gut microbiota.
	Zhang et al. (2021) (19)	C57BL/6 mice (6–8 weeks old, male, weighing 20 g)	ICH model was induced by 0.03 U collagenase.	Spontaneous bacterial infection	Significant immunosuppression of the peripheral immune system was observed at 3 day but improved at 7 day following hemorrhagic stroke; intestinal barrier function↓; immune disorders↑; Intestinal bacteria had migrated to lung tissue and caused lung infection on the 7th day after hemorrhagic stroke.	Reveal the dynamic process of infection after hemorrhagic stroke and provide clues for the optimal timing of intervention for secondary pulmonary infection in stroke patients.
	Diaz-Marugan et al. (2023) (43)	Male wild-type C57BL/6 mice aged 11 to 12 weeks. Cx3cr1^eGFP/+^ (CX3C motif chemokine receptor 1) male and female mice	MCAO for 45 minutes	Spontaneous bacterial infection	Lymphocytopenia; widespread colonization of lung and other organs by opportunistic commensal bacteria; gut epithelial barrier resistance↓; complement and nuclear factor-κB activation; gut regulatory T cells↓; a shift of gut lymphocytes to γδT cells and T helper 1/T helper 17 phenotypes; conjugated BAs in the liver↑; BAs and SCFAs in the gut↓; Gut fermenting anaerobic bacteria ↓; Opportunistic facultative anaerobes, notably Enterobacteriaceae↑	Stroke facilitates a bloom of opportunistic commensals in the gut microbiota. However, this bacterial expansion in the gut does not mediate poststroke infection.
Changes in immune molecules or signaling pathways	Schulte-Herbrüggen et al. (2008) (44)	C57/Bl6 mice; 10-12-week-old male mice weighing 18–24 g	tMCAO for 60 min	Spontaneous pneumonia	Immunomodulatory neuropeptide α-MSH in the lung↑; production of IL-10 by lung macrophages↑; pulmonary lymphocyte counts ↓; lymphocytic IFN-γ↓; lymphocytic IL-4 ↑.	α-MSH plays an important role in the increased infectious susceptibility after cerebral ischemia.
	Ritzel et al. (2019) (45)	CD200R1^−/−^ mutants and CD200R1^+/+^ wild-type littermates; Young adult male mice (12–14 weeks of age).	tMCAO for 60 min	Spontaneous lung infection	Early after ischemia (72 h), CD200R1-KO mice had significantly poorer survival rates and an enhanced susceptibility to spontaneous bacterial colonization of the respiratory tract compared to WT controls; the CNS inflammation was resolved by day 7 post-stroke in WT mice, brain-resident microglia and monocyte activation persisted in CD200R1-KO mice, accompanied by a delayed, augmented lymphocyte response; CD200R1-KO mice displayed greater weight loss, more severe neurological deficits, and impaired motor function compared to WT. Systemically, CD200R1-KO mice exhibited signs of persistent infection including lymphopenia, T cell activation and memory conversion, and narrowing of the TCR repertoire.	CD200-CD200R1 inhibitory signaling prevents spontaneous bacterial infection and promotes resolution of neuroinflammation and recovery after stroke.
	Jin et al. (2019) (46)	C57BL/6 mice	tMCAO for 60 min	Spontaneous lung infection	CD147 expression in the lung, circulating platelets and leukocytes↑; the stroke-associated lung histological damages↑; bacterial load↑; vascular permeability↑; pulmonary edema↑.	Inhibition of CD147 ameliorates aberrant lung inflammatory and immune response and reduces bacterial infection after stroke.
Cerebral infarct volume and infection risk	Liesz et al. (2009) (22)	Sexually mature male C57BL/6 mice (10 to 12 weeks of age)	Coagulation Model (permanent MCAO by distal coagulation); Filament Models (tMCAO for 30 or 90 min)	Spontaneous lung infection	Extensive infarcts (vs. small infarcts): Induced leukopenia at 24h, 3d, and 7d after MCAO; lymphocyte counts in spleen, lymph nodes, and thymus↓; splenic lymphocyte apoptosis↑; blood cytokine production↑; Caused hypothermia and weight loss; pneumonia and sepsis↑; Small infarcts: No significant change in differential blood count; No reduction of overall cell counts in lymphatic organs.	In contrast to infarct size, location and side of the infarct did not affect physiological parameters and immune cell alterations.
	Shim et al. (2019) (18)	Adult male C57Bl/6 mice of 7–12 weeks; 7–8-week-old male C57Bl/6J mice.	tMCAO for 30 or 60 min; permanent MCAO	Spontaneous lung infection	At 1 day following stroke onset, the proportion of mice with infection was significantly greater in mice that had larger infarct sizes; the presence of lung infection in these mice with severe strokes extended past 2 days, suggestive of long-term immune impairment; the elevated risk of infection in more severe stroke is associated with reduced cellularity in peripheral blood, owing predominately to markedly decreased lymphocyte numbers; lymphocyte-to-neutrophil ratio in the lung↓; the location of cerebral infarct does not impact on the susceptibility of post-stroke infection.	Stroke severity, and not infarct location, influences the risk of infection after stroke.
	Wen et al. (2019) (47)	Young (7–10 weeks) and older (12–15 months) male C57BL/6J mice.	tMCAO for 20 min	10^10^ colony forming units of the streptomycin‐resistant E. coli strain DLL206 was inoculated into mice via oral gavage 3 hr after MCAO surgery.	Older mice (>12 months) present with greater spontaneous bacterial lung infections compared to their younger counterparts (7–10 weeks) after stroke; older poststroke mice exhibited elevated intestinal inflammation and disruption in gut barriers following stroke, including reduced expression of mucin and tight junction proteins. the localized pro-inflammatory microenvironment driven by increased TNF-α production in the colon of older mice facilitates the translocation and dissemination of orally inoculated bacteria to the lung following stroke onset.	Advanced age promotes colonic dysfunction and gut-derived lung infection after stroke.

### 3.3 Immunomodulatory therapies for SAP

Due to the complex immunopathogenesis of SAP, conventional antibiotic therapy faces limitations such as antimicrobial resistance and an inability to reverse immunosuppression. Therefore, modulation of specific immune pathways has emerged as a promising therapeutic direction. This section summarizes immunomodulatory therapies that have shown potential in animal models, categorized according to their respective targets of action.

#### 3.3.1 Suppressing the SNS

Collectively, a substantial body of evidence from animal studies indicates that pharmacological inhibition of excessive SNS activation represents a potentially effective strategy for reversing post-stroke immunosuppression and preventing SAP. As a key initiating factor in post-stroke immune dysregulation, the SNS has emerged as an important therapeutic target for neuro-immune modulation.

The non−selective β−blocker propranolol represents the most extensively studied intervention. The seminal study by Prass et al. demonstrated that propranolol significantly reduces post-stroke bacterial burden and mortality and further revealed that its protective effect is distinctly dose- and time-dependent (17). Regarding dose dependency, the study showed that only higher doses of propranolol (3×10 mg/kg and 3×30 mg/kg), administered immediately after MCAO, effectively reduced or prevented bacterial dissemination at 72 hours and restored the impaired IFN-γ response measured 12 hours after stroke. In contrast, lower doses had no effect on either bacterial load or IFN-γ production. In terms of time dependency, the research particularly emphasized that early intervention appears crucial. Delaying drug administration until 24 hours post-ischemia (3×30 mg/kg) neither elevated IFN-γ levels nor reduced bacterial load. This indicates that early suppression of sympathetic activation after stroke may be important for preventing lymphocyte dysfunction and subsequent infection. To clarify that the mechanism stems from specific sympathetic inhibition, the study also employed the chemical sympatholytic agent 6-hydroxydopamine (6-OHDA) for validation. This agent selectively destroys sympathetic nerve terminals, reducing tissue catecholamine levels. The results showed that, consistent with propranolol, 6-OHDA pretreatment similarly enhanced IFN-γ production and significantly lowered bacterial loads in the lungs and blood. Taken together, these findings provide evidence that inhibiting sympathetic signaling is an effective approach for preventing post-stroke infection (17). Subsequent studies have identified its protective mechanisms: McCulloch et al. found that it prevents the loss of splenic marginal zone B cells and maintains circulating IgM levels (25); Liu et al. showed that it improves the function of splenic NK cells (26). In a model mimicking aspiration pneumonia, Prass et al. showed that prophylactic administration of propranolol prevents the development of pneumonia following bacterial inhalation (24).

However, SNS−targeted interventions exhibit target specificity and dual effects. For instance, Wong et al. reported that the protective effect of propranolol depends on liver iNKT cells, as it was ineffective in iNKT−cell−deficient mice, indicating that its action requires mediation by specific immune cells (16). The efficacy of SNS−targeted interventions varies considerably across different agents, and current evidence remains inconclusive regarding the optimal pharmacological approach. Zhou et al. found that the norepinephrine−release inhibitor dexmedetomidine did not improve SAP in mice (48). Moreover, Xu et al. reported that treatment with the selective β2−adrenergic receptor agonist clenbuterol reduced bacterial burden and suppressed pneumonia, but concurrently increased cerebral infarct volume, revealing the potential risks associated with targeting specific adrenergic receptor subtypes (49). These disparate outcomes highlight that the translational promise of SNS modulation is tempered by unresolved questions concerning receptor subtype specificity, dosing, and timing, and that findings should be interpreted with caution until further validated across multiple models and laboratories.

In summary, the non−selective β−blocker propranolol has consistently demonstrated protective effects against post−stroke pneumonia in multiple animal studies, supporting the therapeutic rationale for SNS inhibition. However, the variable and occasionally detrimental outcomes observed with other SNS−targeting agents underscore that the efficacy of this approach is highly dependent on the specific pharmacological agent, receptor subtype, and treatment timing. These findings remain predominantly model−dependent, and their translation to clinical practice requires further validation in more representative preclinical models that account for aging and comorbidities.

#### 3.3.2 Suppressing the HPA axis

In interventional studies targeting the HPA axis, the glucocorticoid receptor antagonist RU486 has been the primary agent explored. Available animal evidence indicates that blocking the HPA axis can ameliorate specific post-stroke immune cell deficiencies; however, its role in preventing systemic infection appears limited, suggesting that its contribution to the post-stroke immunosuppressive network may be subordinate to that of the SNS.

Two studies have evaluated the effects of RU486 from distinct perspectives. Prass et al. found that although RU486 treatment restored the splenocyte apoptosis rate in MCAO mice to sham−surgery levels and prevented peripheral lymphocytopenia, it failed to reduce bacterial loads in the blood or lungs (17). In contrast, concurrent treatment with the β−blocker propranolol significantly alleviated infection and improved survival, suggesting that the SNS may play a more critical role than the HPA axis in driving post−stroke infection susceptibility. Liu et al. provided another perspective, showing that HPA−axis activation contributes to post−stroke splenic atrophy and numerical reduction of peripheral NK cells. Their study revealed that combined administration of propranolol and RU486—blocking both SNS and HPA−axis signaling to NK cells—significantly enhanced NK cell−mediated immune defense against pneumonia (26). These findings suggest that HPA−axis inhibition may have value in restoring the function of specific innate immune cells.

Taken together, these limited but consistent findings indicate that pharmacological inhibition of the HPA axis (e.g., with RU486) can reverse stroke−induced lymphocyte apoptosis and NK−cell depletion, supporting a contributory role of the HPA axis in post−stroke immunosuppression. However, blocking the HPA axis alone appears insufficient to effectively prevent bacterial pneumonia, and its therapeutic significance may lie primarily in the auxiliary restoration of specific immune cell functions within combined immunomodulatory regimens. Further studies are needed to clarify its potential value and to determine whether HPA axis modulation offers meaningful clinical benefit beyond SNS−targeted approaches.

#### 3.3.3 Suppressing the CAP

Intervention in the CAP requires greater caution, as its core challenge lies in balancing the anti-inflammatory benefits of this pathway with the preservation of essential pulmonary antibacterial defenses. Current research has primarily focused on inhibiting its excessive activation in an attempt to restore pulmonary immunity.

Lafargue et al., using a model of stroke combined with *Pseudomonas aeruginosa* pneumonia, found that intervention with methyllycaconitine—a specific inhibitor of the α7nAChR—significantly attenuated the impact of prior stroke on lung injury and mortality caused by *P. aeruginosa* pneumonia (31). Conversely, pretreatment with the α7nAChR agonist PNU−282987 before stroke markedly exacerbated lung injury induced by *Pseudomonas aeruginosa* pneumonia. Mechanistically, this exacerbation was associated with reduced release of the key neutrophil chemokine keratinocyte chemoattractant (KC) and diminished intracellular bacterial killing by both a murine alveolar macrophage cell line and primary murine neutrophils (31). These findings indicate that inhibiting the overactive CAP following stroke may help restore antibacterial immunity.

Overall, initial evidence from a single study suggests that pharmacological suppression of the CAP via α7nAChR antagonism may mitigate SAP−related injury, providing support for the dual−edged nature of this pathway. However, given the unresolved questions regarding nAChR subunit specificity and the reliance on a limited number of experimental models, these findings should be interpreted with caution. Further studies employing diverse pneumonia models and rigorous receptor pharmacology are required before the therapeutic potential of CAP inhibition can be reliably assessed.

#### 3.3.4 Modulation of immune cells

The seminal work by Prass et al. showed that infusion of functional T cells and NK cells significantly reduced post−stroke bacterial burden and mortality, providing evidence that restoring cellular immune function can be sufficient to prevent infection (17). Subsequent efforts shifted toward precisely modulating endogenous immune cells. Wong et al. found that activating liver iNKT cells with α-galactosylceramide reversed stroke-induced systemic immunosuppression and prevented infection, thereby identifying a novel target for immunomodulation based on a specific cell subset (16). Furthermore, pharmacological inhibition of splenic apoptosis has also been investigated as a potential strategy. Jin et al. found that the statin drug simvastatin significantly attenuated post-stroke splenic atrophy and splenic apoptosis by modulating the mitochondrial apoptosis pathway—specifically, by increasing the expression of the anti-apoptotic protein Bcl-2 and reducing the translocation of the pro-apoptotic protein Bax from the cytoplasm to mitochondria. This intervention thereby lowered the susceptibility of mice to spontaneous pulmonary bacterial infection (50). More recently, cell−based therapy has shown promise. Li et al. reported that infusion of bone marrow−derived mesenchymal stem cells (BM−MSCs) markedly reduced pulmonary bacterial burden in stroke mice. Mechanistically, BM−MSCs released migrasomes enriched with the antimicrobial peptide dermcidin, which dually enhanced the function of pulmonary macrophages: exerting a direct antibacterial effect while promoting bacterial clearance through enhanced LC3−associated phagocytosis (51). These findings indicate that BM−MSCs constitute a potential therapeutic approach with dual functions—direct antimicrobial activity and immunomodulation.

Collectively, these diverse studies suggest that direct targeting of dysregulated immune cells—via activation of specific subsets (e.g., iNKT cells), pharmacological inhibition of splenic apoptosis (e.g., simvastatin), or administration of multifunctional stem cells (e.g., BM−MSCs)—represents a promising complementary approach to restoring post−stroke immune homeostasis. However, each strategy has been evaluated in only a limited number of studies, and their relative efficacy, optimal timing, and safety profiles remain to be systematically compared. Further validation in independent laboratories and clinically relevant models is warranted.

#### 3.3.5 Modulation of cytokines and immune signaling pathways

Interventions targeting specific cytokines and signaling pathways offer a diverse array of molecular targets for the immunotherapy of SAP. These strategies aim to modulate the dysregulated immune network following stroke.

Regarding neuropeptide-targeted approaches, Schulte-Herbrüggen et al. reported that the immunomodulatory neuropeptide α-MSH is rapidly up-regulated in the lungs within 24 hours after cerebral ischemia. Systemic administration of agouti, a naturally occurring antagonist of the MC-1R, immediately after MCAO significantly reduced the pulmonary bacterial burden at 72 hours (44). Zhao et al. reported that orexin-A, a hypothalamic-derived neuropeptide, alleviates cerebral ischemic inflammatory injury in mice by suppressing the NF-κB signaling pathway, mitigated stroke-induced immunosuppression, and reduced pulmonary bacterial load in the MCAO model (52).

In the context of cytokine modulation, Prass et al. found that systemic administration of IFN-γ on the first day after stroke significantly reduced bacterial burden (17). In contrast, Jagdmann et al. observed that intratracheal delivery of IFN-γ only modestly improved the pulmonary cytokine profile but failed to prevent spontaneous infection and might even impair bacterial clearance (53). Dames et al. showed that granulocyte-macrophage colony-stimulating factor (GM-CSF) therapy improves peripheral and pulmonary leukocyte counts, enhances the peripheral cytokine response, lowers early pulmonary bacterial burden, and improves long-term functional outcomes after experimental stroke (54). Zhang et al. found that while interleukin-33 (IL-33) therapy provided neuroprotection by reducing brain inflammation, it unexpectedly exacerbated systemic immunosuppression and worsened pulmonary infection; however, the study suggested that combining IL-33 with antibiotics could retain its neuroprotective benefits while mitigating infection risk (55). Zhang et al. reported that interleukin-37 (IL-37) in transgenic mice concurrently attenuated brain injury and reduced bacterial pulmonary infection (56).

Concerning interventions targeting cell surface molecules and signaling pathways, Jin et al. showed that anti-CD147 antibody treatment alleviated post-stroke lung injury and infection, primarily by modulating the expression of key pulmonary cytokines. Mechanistically, the antibody reduces IL-17A expression in pulmonary γδ T cells, thereby decreasing inflammatory cell infiltration and mitigating lung tissue damage, while also enhancing IFN-γ expression in pulmonary NK1.1^+^ cells and CD4^+^ T cells, leading to a reduction in bacterial load (46). Roth et al. found that post-stroke administration of the caspase-1 inhibitor VX765 improved T cell survival. In subsequent pneumonia models induced by *Streptococcus pneumoniae* or *Klebsiella pneumoniae*, VX765 treatment reduced the pulmonary bacterial load in stroke mice to levels comparable to sham-operated controls, whereas control stroke mice exhibited significantly elevated bacterial loads. These results suggest that inflammasome inhibition may enhance post-stroke immune function in part by rescuing T cell death, thereby reducing post-stroke pulmonary infection (34). McCulloch et al. reported that treatment with IgM-enriched intravenous immunoglobulin (IgM-IVIg) effectively replenished IgM depleted after stroke, improved the clearance of stroke-associated bacterial pulmonary infection, and did not affect cerebral infarct volume (57).

In summary, interventions targeting cytokines and immune signaling pathways represent a diverse and conceptually attractive class of strategies for SAP. However, the current evidence base is highly fragmented, with most individual targets having been evaluated in only one or two studies. Moreover, the divergent outcomes observed with the same molecule under different conditions—for instance, the contrasting effects of IFN−γ depending on route of administration, and the trade−off between neuroprotection and worsened infection with IL−33—underscore the context−dependent nature of cytokine modulation. At present, these findings should be viewed as exploratory, and their translation into clinical applications will require independent replication, systematic comparison of different strategies, and a deeper understanding of the optimal timing, route, and combination regimens that can achieve anti−infective benefits without compromising neurological recovery.

#### 3.3.6 Modulation of gut-lung axis

Overall, modulating the “gut–lung axis” to intervene in post-stroke intestinal barrier and gut microbial homeostasis has been proposed as a novel approach for the prevention and treatment of SAP. Emerging research has provided experimental support for this concept. Miao et al. found that the herbal formula GCis (Ginseng Radix et Rhizoma, Aconiti Lateralis Radix Praeparata, and Cistanches Herba) could prevent pulmonary infection following ICH by enhancing peripheral immunity (e.g., increasing splenic and thymic indices, leukocyte and lymphocyte counts) and strengthening the intestinal mucosal immune barrier (58). Wang et al. showed that the herbal formula Tongfu Xingshen capsule (TFXS) not only alleviated brain injury and neuroinflammation but also significantly improved pneumonia and lung injury, with this multi-organ protective effect being dependent on the gut microbiome. Mechanistically, TFXS modulated stroke-induced gut dysbiosis, reversed associated sphingolipid metabolism disorders and ceramide accumulation, and increased the abundance of probiotics such as *Lactobacillus*, *Allobaculum*, and *Enterococcus*. Moreover, it repaired the intestinal barrier by increasing acidic mucus secretion and the expression of the tight-junction protein zonula occludens-1 (ZO-1). These effects collectively produced synergistic anti-inflammatory and protective outcomes in the brain, lungs, and gut (59).

In summary, preliminary studies on herbal formulas have provided initial evidence that targeting the gut–lung axis may offer a novel, multi−mechanism approach for SAP prevention. However, the current evidence is limited to two herbal interventions, each evaluated in a single study, and the observed effects have yet to be independently replicated. Moreover, the specific active constituents responsible for the beneficial effects, and the causal relationship between gut microbiota modulation and pneumonia outcomes all remain to be established. At present, the gut–lung axis represents an intriguing emerging concept rather than a validated therapeutic target, and rigorous further investigation in diverse preclinical models with direct pneumonia endpoints is essential to determine its translational potential. Immunomodulatory therapies for treating SAP in animal studies are summarized in **Table 2**.

**Table 2:** Immunomodulatory therapies for treating SAP in animal studies. Experimental animal studies are represented in this table.

Therapeutic mechanism	Author (year)	Animal	model	Intervention methods	Primary outcomes	The impact on neural function
Inhibition of SNS	Prass et al. (2003) (17)	Gender-mixed 7-wk-old or older SV129/J mice or C57BL/6J mice weighing 18–22 g; B6.129P2-Tcrb^tm1Mom^ (αβ T cell–deficient), B6.129P2-Tcrd^tm1Mom^ (γδ T cell–deficient), B6.129S7-INFγ^tm1Ts^ (IFN-γ–deficient).	tMCAO for 60 min	Propranolol (i.p., 1–30 mg/kg body weight as indicated, immediately before as well as 4 and 8 h after MCAO. Where indicated, propranolol administration (30 mg/kg body weight) was delayed and given 24, 28, and 32 h after MCAO); 6-OHDA (i.p., 200 mg/kg body weight, 3 d before MCAO).	Poststroke lymphocyte dysfunction (i.e., decrease of IFN-γ and increase of IL-4 production)↓; Bacterial infections↓; Mortality (propranolol)↓.	/
	Prass et al. (2006) (24)	129S6SvEv mice (aged 9–13 weeks, mixed gender)	tMCAO for 60 min; Aspiration pneumonia (intranasal application of 20 microL of a defined suspension of *Streptococcus pneumoniae* in PBS 4 or 14 days after MCAO)	Propranolol (30 mg/kg body weight, immediately before as well as 4 and 8 hours after MCAO)	Pneumonia↓; bacteremia↓.	/
	Wong et al. (2011) (16)	Balb/c and CD1d-deficient (Cd1d^-/-^, Balb/c background) mice; Cxcr6^gfp/+^ mice; male mice of 8–12 weeks old.	tMCAO for 30 min	Propranolol or 6-OHDA (24 hours after MCAO)	Infections after stroke↓; Mortality↓; phenotypic changes of iNKT cells↓; the effects of propranolol were entirely dependent on iNKT cells; reversed the preference for intracellular IL-10 production back to an intracellular IFN-γ dominant production and toward a Th1-dominant phenotype.	No effect on infarct size.
	McCulloch et al. (2017) (25)	Male 8–10-week-old C57BL/6J mice; Ccr2^RFP/+^ transgenic mice	tMCAO for 40 min	Propranolol (i.p., 30 mg/kg, immediately before and 4 h after MCAO)	Loss of splenic marginal zone B cells↓; IgM levels↑; bacterial burden↓;	/
	Liu et al. (2017) (26)	Adult male 10-to 12-week-old mice Male C57BL/6; Rag2^-/-^Il2rg^-/-^; nAChR β2-subunit genetically ablated (Chrnb2^-/-^) mice; Cd1d^-/-^NK1.1-tdTomato mice	tMCAO for 60 min	Propranolol (i.p., 30 mg/kg, immediately after MCAO) + RU486 (i.p., 30 mg/kg body weight immediately after MCAO)	NK cell-mediated immune defenses↑; post-stroke infection↓.	/
	Xu et al. (2022)b (49)	8–10 weeks old male C57BL/6 mice (24–26 g)	tMCAO for 60 min	Selective β2-adrenergic receptors agonist clenbuterol (i.p., 1 mg/kg, 24 h and 48 h after MCAO)	NE levels at 3 days after stroke↓; percentage of total T cells and T helper cells↓; percentages of cytotoxic T cells↑; percentage of B cells↓; IL-4, IL-10, and TGF-β1↑; TNF-α, IL-1β, and IFN-γ↓; percentage of Gr-1+ neutrophils and B cells↓; percentage of NK cells↑; bacterial burden↓; pneumonia↓; TNF-α expression in lung↓	Infarct volume↑.
	Zhou et al. (2023) (48)	Male C57BL/6 mice weighing 22–27 g at 8–10 weeks	tMCAO	Dexmedetomidine, an inhibitor of NE (i.p., 25 μg/kg, P1 (three times daily, every 2 h since 24 h after reperfusion) and P2 (once daily) hours following MCAO)	NE levels↓; not alleviating SAP symptoms; spleen size and spleen index↑; CD3+ T cell population in both the blood and brain↓; CD3+ T cell population in the spleen↑.	Not decreasing the infarct area.
Inhibition of HPA	Prass et al. (2003) (17)	Gender-mixed 7-week-old or older SV129/J mice or C57BL/6J mice weighing 18–22 g; B6.129P2-Tcrb^tm1Mom^ (αβ T cell–deficient), B6.129P2-Tcrd^tm1Mom^ (γδ T cell–deficient), B6.129S7-INFγ^tm1Ts^ (IFN-γ–deficient).	tMCAO for 60 min	RU486 (i.p., 30 mg/kg body weight) 24 h, 5 h, and immediately before MCAO)	Peripheral blood lymphocyte counts↑; the percentage of apoptotic splenocytes↓; no effect on blood or pulmonary bacterial burden.	/
	Liu et al. (2017) (26)	Adult male 10-to 12-week-old mice Male C57BL/6; Rag2^-/-^Il2rg^-/-^; nAChR β2-subunit genetically ablated (Chrnb2^-/-^) mice; Cd1d^-/-^NK1.1-tdTomato mice	tMCAO for 60 min	RU486 (i.p., 30 mg/kg body weight immediately after MCAO) + Propranolol (i.p., 30 mg/kg, immediately after MCAO)	NK cell-mediated immune defenses↑; post-stroke infection↓.	/
Inhibition of CAP	Lafargue et al. (2012) (31)	C57BL/6 mice; Male mice aged 10-to 14-week-old; α7-knockout mice	tMCAO for 60 min; followed by 24 h of reperfusion, then 50 μL of PBS containing 1×10^7^ CFU of *Pseudomonas aeruginosa* was instilled into both lungs through the trachea via the mouth	Methyllycaconitine, a specific inhibitor of the α7nAChR (i.p., 10 mg/kg, 1 h before MCAO)	The effect of prior stroke on lung injury↓; mortality caused by *P. aeruginosa* pneumonia↓.	No effect on the brain infarct area or neurological score after MCAO.
Modulation of cells	Wong et al. (2011) (16)	Wild type mice; mice deficient in iNKT cells (Cd1d^–/–)^; Cxcr6^gfp/+^ mice	MCAO	α-galactosylceramide, a specific activator of iNKT cell (24 hours after MCAO)	Systemic levels of endogenous IFN-γ↑; stroke-induced neutrophil pulmonary influx↓; infections after stroke↓.	No notable differences in infarct sizes.
	Jin et al. (2013) (50)	Male C57BL/6J mice (8 to10 weeks old, 22 to 25g)	tMCAO for 60 min	Simvastatin (s.c., 20 mg/kg/day, for 5 days, with the first dose given immediately after MCAO)	Spleen atrophy and splenic apoptosis↓; mitochrondrial antiapoptotic Bcl-2 expression↑; proapoptotic Bax translocation from cytosol into mitochondria↓; brain IFN-γ (3 days after stroke) ↓; the stroke-associated lung susceptibility to spontaneous bacterial infection↓.	infarct volume and neurological deficits (5 days) after stroke↓;
	Li et al. (2023) (51)	C57/Bl6 wild-type mice; Male mice that were 8–10 weeks old with a weight of 18–25 g	tMCAO for 60 min	BM-MSC (i.v., 2×10^6^ cells per mouse, 2 h after reperfusion)	Survival rate at 1–14 d after stroke↑; Bacterial load in the lungs↓; the bacterial phagocytosis of pulmonary macrophages↑; pulmonary inflammatory resolution↑.	Neurological deficit scores↓; Infarct volume↓.
Modulation of cytokines and immune signaling pathways	Prass et al. (2003) (17)	Gender-mixed 7-week-old or older SV129/J mice or C57BL/6J mice weighing 18–22 g; B6.129P2-Tcrb^tm1Mom^ (αβ T cell–deficient), B6.129P2-Tcrd^tm1Mom^ (γδ T cell–deficient), B6.129S7-INFγ^tm1Ts^ (IFN-γ–deficient).	tMCAO for 60 min	Recombinant IFN-γ (i.p., 2 μg, at 24 and/or 48 h after MCAO)	Bacterial dissemination in peripheral blood and lungs↓; (24 h, but not 48 h, after cerebral ischemia)	/
	Schulte-Herbrüggen et al. (2008) (44)	C57/Bl6 mice; 10–12 week old male mice weighing 18–24 g	tMCAO for 60 min	Agouti, MC-1R antagonist (i.v., 3×1 mg/kg, immediately after MCAO as well as 6 and 12 h after MCAO)	Pulmonary bacterial burden at 72 h↓;	No significant influence on the infarct size.
	Dames et al. (2018) (54)	Male C57Bl/6J mice	tMCAO for 60 min	Recombinant mGM-CSF (s.c., 10 μg in 100 μl PBS, 6, 30 and 54 h after MACO)	Peripheral and pulmonary leukocyte numbers↑; Peripheral cytokine responses↑; lung bacterial burden in the early course↓.	Long-term functional outcome↑.
	Zhang et al. (2018) (55)	Male C57BL/6 mice aged 8–12 weeks; male double reporter IL-10^eGFP^Foxp3^mRFP^ mice aged 12–16 weeks; male T-bet–deficient (T-bet^-/-^) mice aged 8–12 weeks	tMCAO for 60 or 45 min	Recombinant mouse IL-33 (I.P., 2 μg, 24 hours before and immediately after reperfusion into C57BL/6 and T-bet^-/-^ mice); Recombinant mouse IL-33 (I.P., 0.4 μg, 24 hours before and immediately after reperfusion into IL-10^eGFP^Foxp3^mRFP^)	Plasma levels of Th2-type cytokines↑; Proinflammatory (3-nitrotyrosine+F4/80+) microglia/macrophages in the brain↓; activated microglia and infiltrating cytotoxic (natural killer-like) T cells↓; IL-10-expressing regulatory T cells↑; lung bacterial infection↑; mortality at 24 hours↑; lower dose of IL-33 did not exacerbate the systemic effects of stroke (i.e., lung infection, latency to fall in hanging wire test, clinical score) while tending to exert a level of neuroprotection similar to that of the higher dose.	Infarct size↓; functional deficits↑.
	Zhang et al. (2019) (56)	Homozygous mice transgenic for human IL-37 (IL-37tg) male mice; C57Bl/6 male mice	tMCAO for 60 min	IL-37tg	Bacterial lung infection↓; pro-inflammatory microglia-macrophages↓; anti-inflammatory markers↑.	Locomotor deficit↓; cerebral infarcts↓;
	Jin et al. (2019) (46)	C57BL/6 mice	tMCAO for 60 min	A rat anti-mouse CD147 monoclonal antibody (tail vein injection, 1 mg/kg, in the 24-h survival experiments, a single dose of antibody was given at 4 h after onset of ischemia; in the 72-h survival experiments, antibody treatment was initiated at 4 or 8 h, and repeated at 24 and 48 h after onset of ischemia)	Lung histological damages↓; bacterial load↓; vascular permeability and pulmonary edema↓; deceased lung inflammatory cell infiltration by reducing IL-17A expression in lung γδ T cells and attenuated bacterial load by enhancing IFN-γ expression in the lung NK1.1+ cells and CD4+ T cells; enhanced platelet-leukocyte aggregates↓;	/
	Jagdmann et al. (2021) (53)	12 weeks old male C57Bl/6 J	tMCAO for 60 min; Nasal inoculation with *S. pneumoniae* on day 4 after experimental stroke	IFN-γ	Bacterial clearance of aspirated pneumococci↓.	/
	Zhao et al. (2021) (52)	Male C57BL/6 J mice aged 8–10 weeks (weighing 22–25 g)	tMCAO for 60 min; Intra-tracheal inoculation with S. pneumoniae (20 microlitres of *S. pneumoniae* suspension containing 2×10^7^ CFU)	Orexin-A (2 nmol, intracerebroventricularly administered over 10 min into the right lateral ventricle 30 min after MCAO)	Bacterial burden in the lung↓; immunodepression↓; microglial infiltration into the brain ischemic penumbra↓; the activation of the NF-κB signaling pathway↓; the translocation of NF-κB p65 from cytoplasm to nucleus in microglial↓;	Brain infarct volume↓; neurological function at 72 h after MCAO↑.
	Roth et al. (2021) (34)	C57BL6/J mice; Aim2^-/-^ mice; Rag-1^-/-^ mice; Casp1^-/-^ mice The ASC-Citrine reporter mice; Pycard^-/-^ mice; Myeloid-specific ASC-deficient mice; Cx3Cr1^GFP/+^ mice Fas^lpr^ mice.	tMCAO for 60 min; The inoculum (10^6^ CFU of *S. pneumoniae* or 2x10^5^ CFU of *K.pneumoniae*) was administered with a pipette onto the nostrils of the mice.	VX765 (caspase-1 inhibitor)	T cell survival ↑; post-injury bacterial load↓.	/
	McCulloch et al. (2022) (57)	Male 8–12-week-old C57BL/6 J mice	tMCAO for 30 min	IgM-IVIg (intravenously via the tail vein, 250 mg/kg body weight, the first dose at 8 AM prior to MCAO or sham surgeries, which were carried out throughout the morning, and again at 24-h post-surgery)	bacterial clearance from the lung↑; lung pathology↑; splenic plasma B cell numbers↑; endogenous mouse IgM and IgA circulating immunoglobulin concentrations↑; elevation of selected pro-inflammatory cytokines in the lung↓; phagocytosis of *Staphylococcus aureus* bioparticles *in vitro*↑.	No effect on brain infarct volume.
Modulation of gut-lung axis	Miao et al. (2022) (58)	Male C57BL/6J mice (7–8 weeks old, 20–24 g)	ICH model (microinfusion pump-mediated injection of 0.03U collagenase type IV-S in 0.5 μLsalineat a constant rate of 0.2 μL/min)	Gcis (Ginseng Radix et Rhizoma, Aconiti Lateralis Radix Praeparata, and Cistanches Herba) (intragastrically administered, 1.3 or 3.9 g/kg/d, once daily for 3 days before ICH surgery and continued for 7 days after ICH surgery)	lung bacterial biomass ↓; pathological abnormalities↓; spleen and thymus indexes, WBC, and LY% contents↑; intestinal mucosal immune barrier↑.	/
	Wang et al. (2025) (59)	Male SD rats (200–240 g; male SD rats (180–200 g);	An aspiration-induced *Klebsiella pneumoniae* infection-complicating ICH model; an intratracheal injection of LPS-induced acute lung injury-complicating ICH model	Tongfu Xingshen capsule (TFXS), (intragastrically administered, 0.5, 1, or 2 g/kg, once daily for 4 consecutive days following Kp inoculation)	Pneumonia and pulmonary injury↓; the infiltration of leukocytes and lymphocytes↓; the infiltration and overactivation of neutrophils↓; intestinal lesions and barrier damage↓; sphingolipid metabolism disorders↓; ceramide accumulation↓; gut microbiota dysbiosis↓; the abundance of probiotics, including Lactobacillus, Allobaculum and Enterococcus↑.	Neurological deficits↓; hematoma absorption↑; brain damage and neuroinflammation↓.

In the **primary outcomes and the impact on neural function** columns, all the results are referred to the animals treated with the immunomodulator agent in comparison with their respective non-treated controls.

### 3.4 Assessment of risk of bias

**Figure 4:**
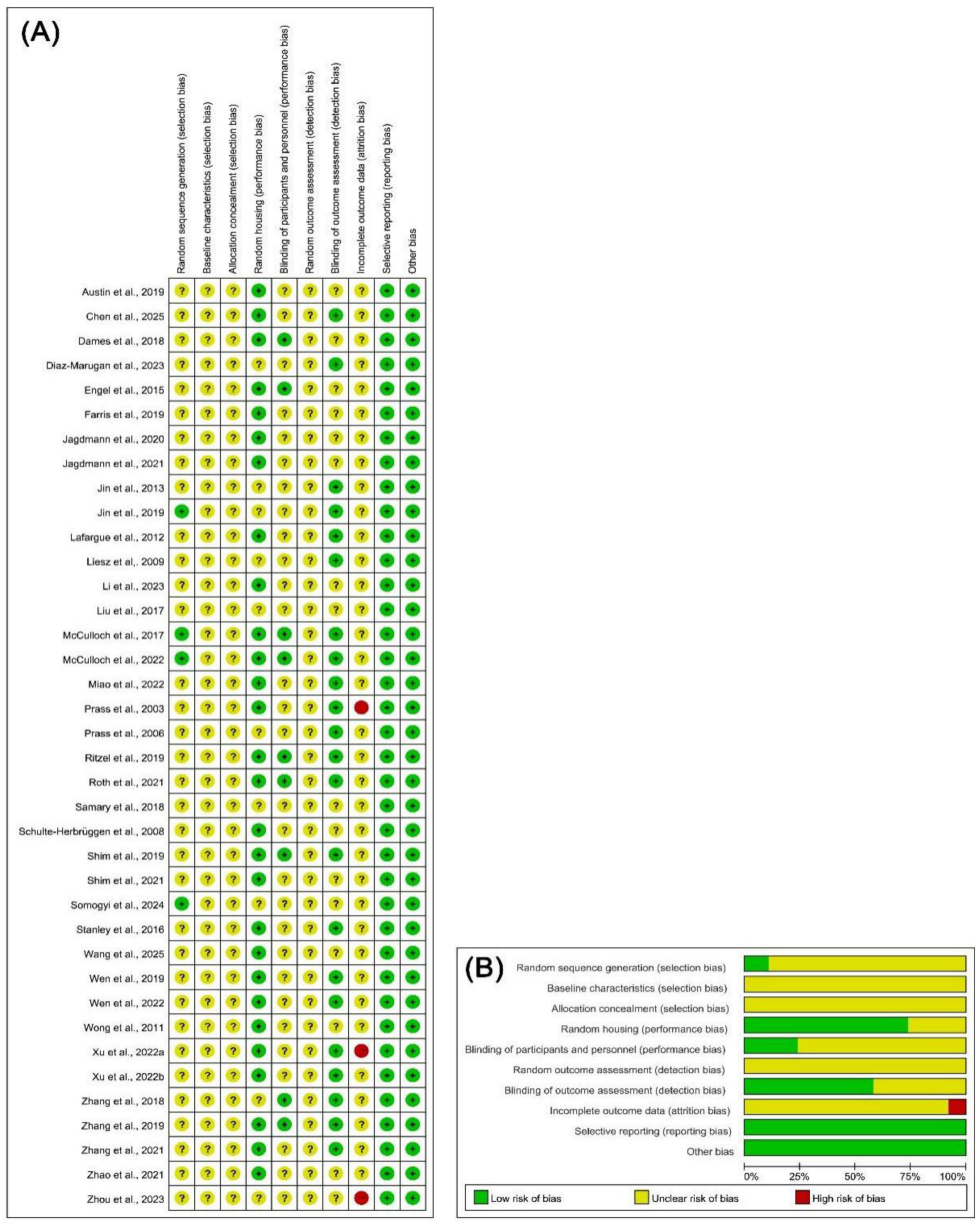
Methodological quality evaluation of bias risk. **(A)** Risk of bias summary, presenting the authors’ judgments for each bias domain across all included studies. **(B)** Risk of bias graph, illustrating the proportion of studies classified as having low, unclear, or high risk of bias for each methodological item.

As shown in **Figure 4**, a methodological quality assessment was performed on the 38 included studies. Overall, the studies demonstrated moderate methodological quality. With regard to selection bias, most studies were judged to have an unclear risk in the domains of “random sequence generation,” “baseline characteristics,” and “allocation concealment,” primarily due to insufficient reporting of methodological details. Concerning performance bias, approximately 75% of the studies showed a low risk in “random housing,” whereas more than half were rated as unclear in “blinding of participants and personnel.” In terms of detection bias, all studies lacked information on “random outcome assessment.” However, over half were assessed as low risk in “blinding of outcome assessment.” For attrition bias, three studies were rated as high risk, while the remainder were unclear. Notably, all studies demonstrated low risk in both reporting bias and other bias. Despite these methodological limitations, particularly the inadequate reporting of randomization and blinding details, all included studies provided valuable core data related to SAP mechanisms or interventions, supporting the qualitative synthesis of this review.

## 4 Discussion

Beyond secondary brain injury mediated by neuroinflammation, post-stroke infections, particularly pneumonia, constitute a pivotal determinant of patient prognosis. SAP affects up to 10% of stroke patients and is strongly associated with increased mortality, worsened neurological outcomes, and a heightened healthcare burden (4, 60). This systematic review synthesizes evidence suggesting that SAP pathogenesis may be a multi-tiered process, centered on a profound disruption of immune homeostasis following stroke. While animal models have elucidated a spectrum of potential immunomodulatory targets, a considerable translational gap persists. This discussion will therefore critically evaluate the alignment between preclinical findings and clinical reality, focusing on: (i) the consistency and complexity of post-stroke immune dysregulation, and (ii) the translational challenges and future directions for immunomodulatory strategies.

### 4.1 Post-stroke immune dysregulation: preclinical and clinical evidence

A cornerstone mechanism revealed across species is the stroke-induced SNS overactivation, which drives a cascade of immunosuppressive events. Animal studies consistently show that post-stroke catecholamine release induces splenic lymphocyte apoptosis (17), impairs hepatic iNKT cell function (16), and depletes splenic B and NK cells (25, 26). Interventions with β-blockers like propranolol reverse these defects and reduce pneumonia risk in models (24). This paradigm is supported clinically: acute stroke patients rapidly develop lymphopenia and monocyte dysfunction (61), while elevated plasma cortisol and adrenaline have been correlated with infection risk and reduced circulating T cell counts (62, 63). Furthermore, the functional consequence of this immunosuppression—diminished IFN-γ production—is evident in both animal models (17, 64) and patients (65). The importance of IFN-γ is underscored by evidence that its early administration reduces bacterial burden in animals (17), and low circulating levels predict infection in patients (66). At the organ level, this neurogenic signal translates into rapid splenic atrophy via catecholamine-mediated apoptosis, observed in animals by day 4 post-stroke (64, 67) and also reported in acute stroke patients via imaging (68). Sustained splenic atrophy is linked to poor prognosis (69), highlighting the SNS-spleen axis as a key pathway linking brain injury to impaired systemic immunity.

However, despite compelling mechanistic synergy, the clinical therapeutic translation of targeting the SNS remains contentious and exemplifies the animal-human translation gap. Although some retrospective analyses suggest β-blockers may reduce SAP incidence and mortality (70), other cohort studies and a prospective trial have failed to confirm this benefit (71–73). Notably, a large cohort study even associated β-blocker use with higher 30-day mortality despite reducing urinary tract infection risk, hinting at complex, infection-specific effects (71). At present, the clinical evidence regarding β-blocker efficacy in SAP remains inconclusive, and the contradictory findings may be partly attributable to differences in study design, patient populations, and treatment protocols that have not been systematically controlled across investigations. These contradictions highlight that the reductive “block-the-SNS” approach derived from animal models fails to capture the clinical complexity, where patient comorbidities, timing, and receptor subtype specificity may critically influence outcomes. The apparent discrepancy between preclinical promise and clinical uncertainty should not be prematurely resolved; rather, it underscores the need for future clinical research to move beyond binary efficacy questions to define the precise patient phenotypes and therapeutic windows that might benefit from finely tuned adrenergic modulation.

In the context of SAP, the HPA axis plays a contributory but subordinate role within the broader neuroimmune response. Its activation leads to glucocorticoid oversecretion, inducing lymphocytopenia and contributing to splenic NK cell depletion (17, 26). Pharmacological blockade with the glucocorticoid receptor antagonist RU486 can reverse these specific cellular defects (17, 26). However, a critical finding is that inhibiting the HPA axis alone fails to reduce post-stroke bacterial infection, in contrast to the efficacy of SNS blockade (17). This suggests that while the HPA axis is an active component of post-stroke immunosuppression, it is not the primary driver of infection susceptibility. Its therapeutic significance may therefore lie not in monotherapy, but as an adjunct within combined immunomodulatory strategies that simultaneously target the more prominent SNS pathway.

The CAP exemplifies the delicate balance required in immunomodulation, presenting a dual role in SAP. While its activation helps limit damaging inflammation (74, 75), excessive CAP activity after stroke paradoxically suppresses essential pulmonary antibacterial defenses, thereby increasing infection risk (31, 32). Clinical observations suggest that increased peripheral cholinergic activity post-stroke correlates with larger infarcts and higher pneumonia risk (76); however, the precise receptor pharmacology underlying this association remains to be fully elucidated. Therefore, while therapeutic targeting of the CAP represents a conceptually attractive strategy, current evidence remains insufficient to determine whether CAP inhibition or activation—and at which receptor subtype—would yield net clinical benefit. These contradictory observations warrant cautious interpretation.

The gut-lung axis has emerged as another critical interface in post-stroke immunology, though its exact role in pulmonary infection remains unresolved and is being actively redefined. Early animal work established that stroke compromises the intestinal barrier, promoting bacterial translocation to the lungs (19, 42). However, more recent evidence challenges the necessity of this translocation for infection, showing that abolishing gut dysbiosis does not prevent lung colonization (43). These contradictory findings remain to be reconciled and may reflect differences in experimental models, stroke severity, and the specific microbial strains investigated. At present, it is unclear whether the gut serves primarily as a source of translocating pathogens or as a modulator of systemic immune-metabolic homeostasis, or both, depending on the experimental context. Further studies that simultaneously track bacterial translocation, immune function, and metabolic profiles are needed to resolve this uncertainty. In parallel, stroke induces a broader gut-immune-metabolic disruption, characterized by reduced intestinal regulatory T cells, a shift of gut lymphocytes toward γδ T cell and Th1/Th17 phenotypes (43), and decreased levels of immunomodulatory metabolites like BAs and SCFAs (43, 77, 78). These observations raise the possibility that the gut’s primary role may be in shaping a permissive systemic immunologic and metabolic milieu, rather than merely being a source of pathogens, though this hypothesis requires direct experimental validation. Clinically, a distinct gut dysbiosis signature (enriched *Enterococcus*, depleted *Prevotella*) linked to specific serum cytokine changes has been identified in SAP patients, providing direct human evidence for this axis (79). While therapies like SCFA supplementation show promise in stroke recovery (80), their efficacy in SAP remains speculative, marking an important avenue for future investigation.

Following the elucidation of these mechanisms, therapeutic strategies targeting the gut-lung axis have begun to emerge, primarily focusing on restoring microbial balance and repairing the intestinal barrier. Notably, our review identified preliminary yet compelling evidence from traditional medicine: two Chinese herbal formulas were found to exert regulatory effects on the post-stroke gut microenvironment, suggesting the potential of multi-component natural products to modulate this complex axis (58, 59). These approaches aim to fortify the intestinal barrier or correct dysbiosis, representing a conceptual shift from pathogen-centric to host- and ecology-centric therapeutic strategies. However, the translation of these gut-directed strategies into clinical applications for SAP prevention faces significant challenges. Unlike the SNS axis, where clinical trials of β-blockers exist (albeit with conflicting results), interventions for the gut-lung axis remain predominantly in the preclinical stage. A major translational hurdle is the unresolved causal ambiguity highlighted by recent research: if gut dysbiosis and bacterial translocation are not decisive factors for infection development (43), then interventions solely aimed at correcting these may have limited efficacy. This necessitates a shift from simply “fixing the gut” to “modulating the systemic immune-metabolic state originating from the gut.” Furthermore, the heterogeneity of human gut microbiota, influenced by diet, antibiotics, and comorbidities, poses a substantial challenge for standardizing therapies such as probiotics or fecal microbiota transplantation (FMT). The preliminary data on herbal formulas also require rigorous validation in well-designed animal studies with infection endpoints before clinical consideration. Therefore, future research must move beyond proving association to establishing causal therapeutic links. Priority should be given to intervention studies with dual endpoints, assessing both the restoration of gut-immune homeostasis and the concrete outcome of pneumonia prevention in animal models that more closely mimic human comorbidities. The exploration of herbal formulas offers a unique opportunity to probe synergistic, multi-target approaches, but it demands mechanistic elucidation of their active components and pathways.

Locally, stroke remodels the pulmonary immune microenvironment, creating a “fertile ground” for infection. Animal studies reveal impaired alveolar macrophage phagocytosis (35), dysfunctional neutrophils retained in lung vasculature (41), and an imbalance in pulmonary immune cell subsets—manifested as a reduction in lymphocytes and an increase in alveolar macrophages and neutrophils (40). Critically, this preclinical finding is reinforced by a key clinical study demonstrating that impaired neutrophil oxidative burst at stroke onset is an independent predictor of subsequent infection (81). Neutrophils, as first responders, exhibit markedly impaired crucial functions—including phagocytosis and mediator secretion—post−stroke (81, 82). Furthermore, clinical studies indicate that a generalized reduction in the numbers of various T cell subsets after stroke is directly associated with an increased risk of secondary infection (83, 84). It should be noted that these immunosuppression-driven vulnerabilities may coexist with direct, non-infectious neurogenic lung injury (e.g., edema, inflammation) (38, 39). This notion is supported by clinical evidence of acute lung injury in stroke patients (85), a combination that significantly complicates both clinical assessment and therapeutic management.

Following the characterization of these local and systemic immune defects, diverse therapeutic strategies aimed at directly restoring immune function have shown preclinical promise. These approaches range from cellular therapies—such as infusing functional lymphocytes or mesenchymal stem cells to replenish depleted populations and enhance antimicrobial activity (17, 51)—to targeted molecular interventions. The latter include activating specific immune subsets (e.g., iNKT cells with α-galactosylceramide) (16), administering cytokines (e.g., GM-CSF) to boost leukocyte numbers and function (54), and targeting key signaling pathways (e.g., inhibiting the AIM2 inflammasome to prevent monocyte-mediated T cell death) (34). However, this very diversity underscores the complexity of clinical translation. For instance, the efficacy of cytokine therapy can be paradoxically dependent on the route and timing of administration, as seen with IFN-γ (17, 53). Similarly, interventions like IL-33 may improve brain outcomes while worsening systemic infection, highlighting the risk of disrupting delicate inter-organ immune balance (55). Therefore, future efforts must move beyond single-target approaches. Exploring rational combination therapies that concurrently address multiple immune defects will be essential to translate these promising mechanisms into effective, personalized SAP prevention strategies.

Finally, this review focused on studies with direct pneumonia endpoints. However, other notable studies, though not assessing pneumonia directly, provide important conceptual insights for future therapy. These include mechanisms like the SNS-β-arrestin2-NF-κB axis in reversible immunosuppression (86), neutrophil-mediated T-cell suppression via arginase I (87), and herbal formula (e.g., Buyang Huanwu Decoction) modulation of AIM2 inflammasome activity to reduce T-cell apoptosis (88). Although these mechanisms have not yet been directly tested in SAP models, they underscore that reversing post-stroke immunosuppression may be feasible through multiple targets, forming a rich foundation for the next generation of adjunctive therapies. However, whether these strategies can prevent pneumonia without exacerbating neuroinflammation remains to be determined.

### 4.2 Challenges in clinical translation

Despite the promising preclinical findings summarized in this review, it is notable that virtually none of the immunomodulatory strategies tested in animal models have successfully translated into clinical practice for SAP prevention or treatment. Several fundamental discrepancies between preclinical models and the clinical reality of stroke likely account for this translational gap: (1) Differences in immune system aging. The overwhelming majority of preclinical studies employ young adult animals (typically 8–12 weeks old), whereas stroke predominantly affects elderly individuals. Immunosenescence—characterized by thymic involution and reduced naïve T-cell output—profoundly alters both baseline immune status and the response to immunomodulatory interventions (89). The few studies that have addressed aging, such as the demonstration by Wen et al. that stroke-induced pulmonary neutrophil dysfunction is aggravated in aged mice, underscore the importance of age as a biological variable (41). Future preclinical studies should incorporate aged animals to better recapitulate the immune environment of the target patient population. (2) Comorbidities commonly present in stroke patients. Clinical stroke populations frequently suffer from hypertension, diabetes, obesity, and atherosclerosis—conditions that independently affect immune function and susceptibility to infection. These comorbidities are almost universally absent in experimental models, which typically employ healthy, genetically homogeneous animals. For example, diabetes is associated with altered neutrophil function and a shift toward pro-inflammatory macrophage phenotypes, which may compromise immune defenses and potentially blunt the efficacy of immunostimulatory therapies (90), while chronic hypertension may alter sympathetic tone and adrenergic receptor sensitivity, modifying the response to β-blockade (91). The absence of these confounding factors in preclinical studies likely contributes to the overestimation of therapeutic efficacy and underestimation of potential adverse effects when interventions are evaluated in heterogeneous patient populations. (3) Timing of intervention. Preclinical protocols typically administer immunomodulatory agents either before stroke onset or within the first few hours post-stroke, a window that is rarely achievable in clinical practice where patients present with variable delays. Moreover, the dynamic evolution of post-stroke immune changes—ranging from early hyperinflammation to subsequent profound immunosuppression—creates distinct therapeutic windows. An intervention that is beneficial during the immunosuppressive phase may be detrimental if initiated during the hyperacute inflammatory phase. The dose- and time-dependent efficacy of propranolol demonstrated by Prass et al., where delayed administration 24 hours post-ischemia was ineffective, clearly illustrates this challenge (17). Clinical translation will require identification of reliable biomarkers to guide individualized timing of immunomodulatory interventions. (4) Safety concerns related to immune activation after stroke. Reversing immunosuppression to prevent infection must be carefully balanced against the risk of exacerbating neuroinflammation and secondary brain injury. Stroke creates a unique state where the blood-brain barrier is compromised and the brain is highly vulnerable to inflammatory damage. Immunostimulatory strategies—such as IFN-γ administration or iNKT cell activation—while reducing bacterial burden, carry the theoretical risk of potentiating cerebral inflammation and worsening neurological outcomes. This is exemplified by the finding that the β2-adrenergic receptor agonist clenbuterol reduced pulmonary bacterial burden but simultaneously increased cerebral infarct volume (49). Conversely, anti-inflammatory approaches targeting the CAP, while potentially limiting neuroinflammation, may further compromise pulmonary antimicrobial defenses. Future therapeutic development should incorporate dual endpoint assessments—evaluating both infection prevention and neurological outcomes—and explore strategies that achieve compartment-specific immunomodulation, such as inhalation-based delivery to target the lungs while minimizing systemic and cerebral effects. (5) Heterogeneity of stroke severity. Preclinical studies typically employ standardized models (e.g., a fixed duration of MCAO) that produce relatively uniform infarct volumes, whereas in clinical practice, stroke severity ranges from minor to devastating. Given that the extent of infarct volume has been correlated with the degree of systemic immunosuppression and infection risk (18, 22), the efficacy of a given immunomodulatory intervention may vary substantially across different severity strata. Patients with severe strokes may require more aggressive immune restoration, while those with minor strokes might be exposed to unnecessary immune-related adverse effects. Future clinical trials should incorporate stratification by stroke severity or utilize predictive biomarkers to tailor immunomodulatory intensity to individual risk profiles. (6) Variability in infection microbiology. The majority of preclinical SAP models utilize a single, well-characterized bacterial strain (e.g., *Streptococcus pneumoniae*, *Pseudomonas aeruginosa*) or purified LPS administered at a controlled time point and dose. In contrast, clinical SAP is caused by a diverse spectrum of pathogens—including Gram-positive and Gram-negative bacteria, anaerobes, and multidrug-resistant organisms—often in the context of microaspiration of oropharyngeal contents. Distinct pathogens engage different immune recognition pathways and possess unique immune evasion mechanisms that may influence the efficacy of host-directed immunomodulatory therapies. Furthermore, the presence of polymicrobial infections and the increasingly recognized role of the respiratory microbiome add layers of complexity not captured in standard models. Addressing this variability will require the development of preclinical models that more faithfully recapitulate the polymicrobial nature of clinical SAP, as well as careful microbiological phenotyping in future clinical studies of immunomodulatory agents.

These translational challenges collectively highlight that the linear extrapolation of findings from young, healthy, genetically identical animals to elderly, comorbid, heterogeneous patient populations with variable stroke severity and diverse infectious etiologies is inherently limited. Addressing these barriers will require the research community to adopt more clinically representative animal models, validate key findings across multiple species and laboratories, and prioritize the development of robust pharmacodynamic biomarkers that can guide clinical trial design. Such efforts are needed to bridge the gap between the promising mechanistic insights obtained from preclinical studies and the development of effective immunomodulatory therapies for SAP patients.

## 5 Conclusions and directions for future research

This systematic review synthesizes current evidence from animal studies, suggesting that the pathogenesis of SAP may be rooted in a multi-layered immune dysregulation network. This network originates from stroke-triggered overactivation of the neuroendocrine system, leading to systemic lymphocyte depletion and functional suppression, and extends to disturbances in the local pulmonary immune microenvironment as well as emerging evidence of dysfunction of the “gut–lung axis.” These findings advance the understanding of SAP mechanisms and provide a conceptual framework for the development of immunomodulatory therapies that go beyond traditional antibiotics.

Based on the above mechanisms, immunomodulatory strategies targeting specific pathways—such as using β-blockers to inhibit sympathetic overactivation, modulating key cytokines, or intervening in the gut microbiota—have shown preventive potential in preclinical research. However, these strategies remain largely experimental, and their translation to clinical practice faces substantial barriers, as discussed in this review. Future studies could focus on the following directions: (1) developing localized immunomodulatory strategies, such as inhalation-based drug delivery, to reduce systemic adverse effects; (2) exploring combination treatment modalities, for example, combining immunomodulators with antibiotics to simultaneously target disease mechanisms and directly eliminate pathogens; and (3) conducting rigorously designed prospective clinical studies in conjunction with reliable biomarkers to identify optimal intervention timing and patient subgroups most likely to benefit. In addition, further mechanistic research is warranted to evaluate multi-target integrative therapies such as those derived from traditional Chinese medicine, although their clinical potential remains to be rigorously assessed.

## 6 Limitations

This systematic review has several limitations. First, our focus on the immunological mechanisms of SAP and corresponding immunomodulatory therapies represents a developing direction within the field. Consequently, there is currently a paucity of targeted clinical randomized controlled trials (RCTs) to validate the efficacy of these strategies. Although we referenced several observational clinical studies in the discussion to enhance clinical relevance, the level of this evidence is low and cannot substitute for conclusions derived from RCTs. Second, all included studies were based on rodent models. While such models are widely used due to their accessibility and suitability for genetic manipulation, inherent differences exist between their immune systems, physiological architecture, and responses to stroke compared to humans, which limits the direct extrapolation of findings to clinical application. Third, significant heterogeneity was observed among the included studies. This heterogeneity is primarily reflected in the type and severity of stroke models (e.g., permanent versus transient middle cerebral artery occlusion models, and differences in infarct volume), the species and inoculum of pathogens used to induce pneumonia, and the methods and timepoints for immunological assessment. Given the substantial heterogeneity among the included studies, a meta-analysis was not feasible. Additionally, the absence of prospective protocol registration limits the *a priori* transparency of our methodological approach, although we adhered rigorously to PRISMA guidelines during the conduct of this review. Despite these limitations, this review provides a foundation for understanding the complex immune mechanisms of SAP and for the future development of therapies by systematically synthesizing the current evidence.

## Data Availability

The original contributions presented in the study are included in the article/**Supplementary Material**. Further inquiries can be directed to the corresponding authors.

## Glossary

6-OHDA: 6-hydroxydopamine
α7nAChR: α7 nicotinic acetylcholine receptor
α-MSH: alpha-melanocyte-stimulating hormone
ACh: acetylcholine
AIM2: absent in melanoma 2
BALF: bronchoalveolar lavage fluid
BAs: bile acids
BM−MSCs: bone marrow-derived mesenchymal stem cells
β2AR: β2-adrenergic receptor
CAP: cholinergic anti-inflammatory pathway
CD200–CD200R1: cluster of differentiation 200–CD200 receptor 1
DMV: dorsal motor nucleus of the vagus
FasL: Fas ligand
FMT: fecal microbiota transplantation
GM-CSF: granulocyte-macrophage colony-stimulating factor
HMGB1: high mobility group box-1 protein
HPA: hypothalamic-pituitary-adrenal
ICH: intracerebral hemorrhage
IFN-γ: interferon-γ
IgM-IVIg: IgM-enriched intravenous immunoglobulin
IL-1β: interleukin-1β
IL-33: interleukin-33
IL-37: interleukin-37
iNKT: invariant natural killer T
KC: keratinocyte chemoattractant
LPS: lipopolysaccharide
MCAO: middle cerebral artery occlusion
MC-1R: α-MSH receptor-1
NF-κB: nuclear factor-κB
NLRP3: NOD-like receptor family pyrin domain-containing protein 3
PVN: hypothalamic paraventricular nucleus
RAGE: receptor for advanced glycation end products
RCTs: randomized controlled trials
SAP: stroke-associated pneumonia
SCFAs: short-chain fatty acids
SNS: sympathetic nervous system
Th1: T helper 1
TLR4: Toll-like receptor 4
ZO-1: zonula occludens 1
