# Supplementary material for: Immunomodulation for stroke-associated pneumonia: a systematic review of mechanistic insight and emerging therapeutic strategies in animal models
Source: Front Immunol. 2026 Jul 16;17:1856660. doi: 10.3389/fimmu.2026.1856660 (PMC13422413; doi:10.3389/fimmu.2026.1856660)

**Supplementary material.** Search strategies in different databases.

## PudMed

((("Stroke"[Mesh] OR "Strokes" OR "Cerebrovascular Accident" OR "Cerebrovascular Accidents" OR "Cerebral Stroke" OR "Cerebral Strokes" OR "Stroke, Cerebral" OR "Strokes, Cerebral" OR "Cerebrovascular Apoplexy" OR "Apoplexy, Cerebrovascular" OR "Vascular Accident, Brain" OR "Brain Vascular Accident" OR "Brain Vascular Accidents" OR "Vascular Accidents, Brain" OR "Cerebrovascular Stroke" OR "Cerebrovascular Strokes" OR "Stroke, Cerebrovascular" OR "Strokes, Cerebrovascular" OR "Apoplexy" OR "CVA (Cerebrovascular Accident)" OR "CVAs (Cerebrovascular Accident)" OR "Stroke, Acute" OR "Acute Stroke" OR "Acute Strokes" OR "Strokes, Acute" OR "Cerebrovascular Accident, Acute" OR "Acute Cerebrovascular Accident" OR "Acute Cerebrovascular Accidents" OR "Cerebrovascular Accidents, Acute") AND ("Pneumonia"[Mesh] OR "Pneumonias" OR "Experimental Lung Inflammation" OR "Experimental Lung Inflammations" OR "Inflammation, Experimental Lung" OR "Lung Inflammation, Experimental" OR "Lung Inflammations, Experimental" OR "Lobar Pneumonia" OR "Lobar Pneumonias" OR "Pneumonias, Lobar" OR "Pneumonia, Lobar" OR "Pneumonitis" OR "Pneumonitides" OR "Lung Inflammation" OR "Inflammation, Lung" OR "Inflammations, Lung" OR "Lung Inflammations" OR "Pulmonary Inflammation" OR "Inflammation, Pulmonary" OR "Inflammations, Pulmonary" OR "Pulmonary Inflammations")) OR ("stroke-associated pneumonia")) AND ("Immunomodulation"[Mesh] OR "Immunomodulations" OR "Immunomodulatory Therapy" OR "Immunomodulatory Therapies" OR "Therapies, Immunomodulatory" OR "Therapy, Immunomodulatory" OR "Immune System"[Mesh] OR "Immune Systems" OR "System, Immune" OR "Systems, Immune" OR "Immune" OR "Immunodeficiency" OR "Immunodepression" OR "Immunosuppressive" OR "immunomodulatory")

## Scopus

( TITLE-ABS-KEY ( ( ( "Stroke" OR "Strokes" OR "Cerebrovascular Accident" OR "Cerebrovascular Accidents" OR "Cerebral Stroke" OR "Cerebral Strokes" OR "Stroke, Cerebral" OR "Strokes, Cerebral" OR "Cerebrovascular Apoplexy" OR "Apoplexy, Cerebrovascular" OR "Vascular Accident, Brain" OR "Brain Vascular Accident" OR "Brain Vascular Accidents" OR "Vascular Accidents, Brain" OR "Cerebrovascular Stroke" OR "Cerebrovascular Strokes" OR "Stroke, Cerebrovascular" OR "Strokes, Cerebrovascular" OR "Apoplexy" OR "CVA (Cerebrovascular Accident)" OR "CVAs (Cerebrovascular Accident)" OR "Stroke, Acute" OR "Acute Stroke" OR "Acute Strokes" OR "Strokes, Acute" OR "Cerebrovascular Accident, Acute" OR "Acute Cerebrovascular Accident" OR "Acute Cerebrovascular Accidents" OR "Cerebrovascular Accidents, Acute" ) AND ( "Pneumonia" OR "Pneumonias" OR "Experimental Lung Inflammation" OR "Experimental Lung Inflammations" OR "Inflammation, Experimental Lung" OR "Lung Inflammation, Experimental" OR "Lung Inflammations, Experimental" OR "Lobar Pneumonia" OR "Lobar Pneumonias" OR "Pneumonias, Lobar" OR "Pneumonia, Lobar" OR "Pneumonitis" OR "Pneumonitides" OR "Lung Inflammation" OR "Inflammation, Lung" OR "Inflammations, Lung" OR "Lung Inflammations" OR "Pulmonary Inflammation" OR "Inflammation, Pulmonary" OR "Inflammations, Pulmonary" OR "Pulmonary Inflammations" ) ) OR ( "stroke-associated pneumonia" ) ) AND TITLE-ABS-KEY ( ( "Immunomodulation" OR "Immunomodulations" OR "Immunomodulatory Therapy" OR "Immunomodulatory Therapies" OR "Therapies, Immunomodulatory" OR "Therapy, Immunomodulatory" OR "Immune System" OR "Immune Systems" OR "System, Immune" OR "Systems, Immune" OR "Immune" OR "Immunodeficiency" OR "Immunodepression" OR "Immunosuppressive" OR "immunomodulatory" ) ) ) AND ( LIMIT-TO ( DOCTYPE , "ar" ) OR LIMIT-TO ( DOCTYPE , "re" ) )


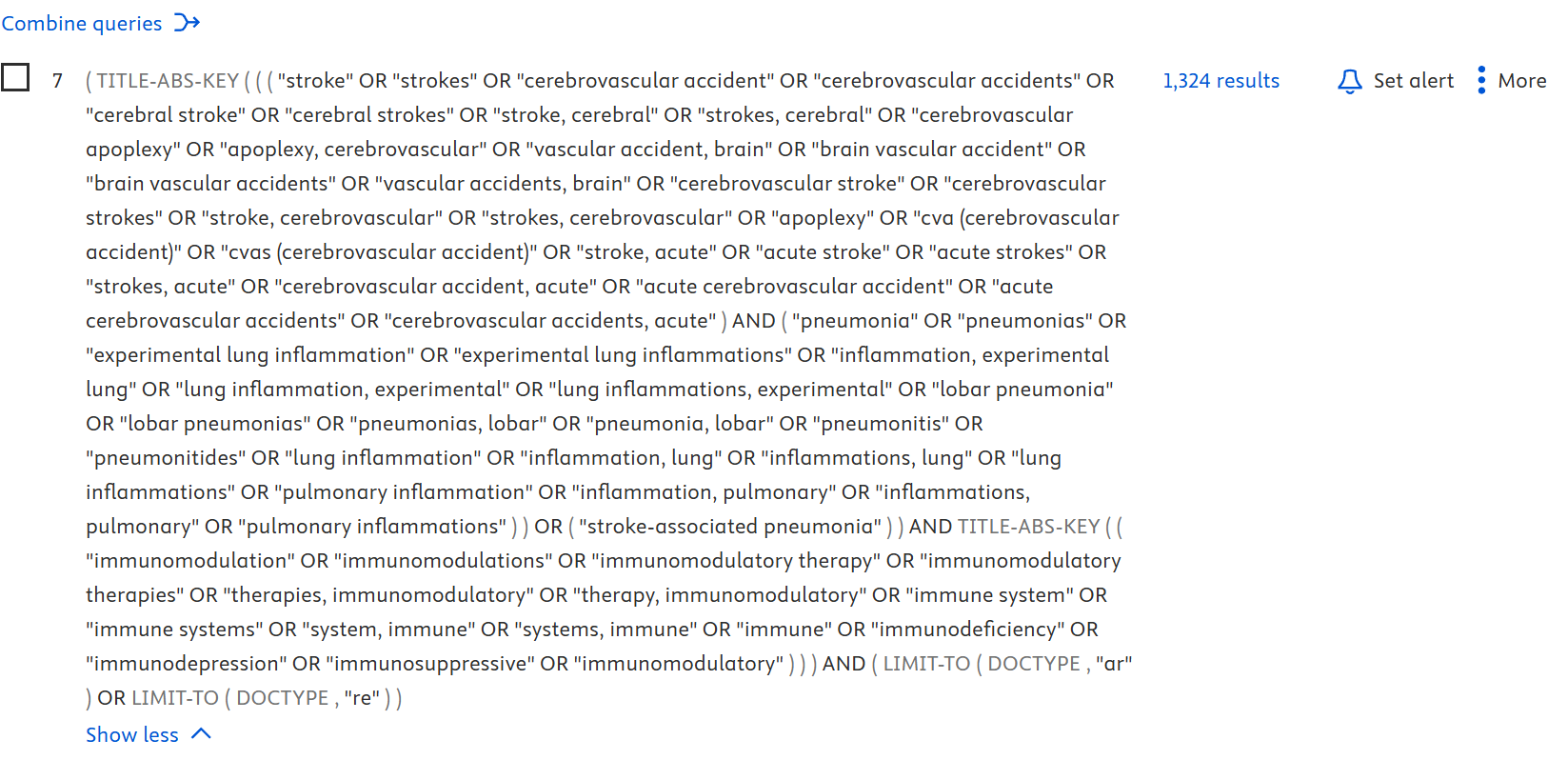


## web of science

(("Stroke" OR "Strokes" OR "Cerebrovascular Accident" OR "Cerebrovascular Accidents" OR "Cerebral Stroke" OR "Cerebral Strokes" OR "Stroke, Cerebral" OR "Strokes, Cerebral" OR "Cerebrovascular Apoplexy" OR "Apoplexy, Cerebrovascular" OR "Vascular Accident, Brain" OR "Brain Vascular Accident" OR "Brain Vascular Accidents" OR "Vascular Accidents, Brain" OR "Cerebrovascular Stroke" OR "Cerebrovascular Strokes" OR "Stroke, Cerebrovascular" OR "Strokes, Cerebrovascular" OR "Apoplexy" OR "CVA (Cerebrovascular Accident)" OR "CVAs (Cerebrovascular Accident)" OR "Stroke, Acute" OR "Acute Stroke" OR "Acute Strokes" OR "Strokes, Acute" OR "Cerebrovascular Accident, Acute" OR "Acute Cerebrovascular Accident" OR "Acute Cerebrovascular Accidents" OR "Cerebrovascular Accidents, Acute") AND ("Pneumonia" OR "Pneumonias" OR "Experimental Lung Inflammation" OR "Experimental Lung Inflammations" OR "Inflammation, Experimental Lung" OR "Lung Inflammation, Experimental" OR "Lung Inflammations, Experimental" OR "Lobar Pneumonia" OR "Lobar Pneumonias" OR "Pneumonias, Lobar" OR "Pneumonia, Lobar" OR "Pneumonitis" OR "pneumonities" OR "Lung Inflammation" OR "Inflammation, Lung" OR "Inflammations, Lung" OR "Lung Inflammations" OR "Pulmonary Inflammation" OR "Inflammation, Pulmonary" OR "Inflammations, Pulmonary" OR "Pulmonary Inflammations")) OR ("stroke-associated pneumonia") (Topic) and ("Immunomodulation" OR "immunomodulation" OR "Immunomodulatory Therapy" OR "Immunomodulatory Therapies" OR "Therapies, Immunomodulatory" OR "Therapy, Immunomodulatory" OR "Immune System" OR "Immune Systems" OR "System, Immune" OR "Systems, Immune" OR "Immune" OR "Immunodeficiency" OR "Immunodepression" OR "Immunosuppressive" OR "immunomodulatory") (Topic) | 341 results

## Embase


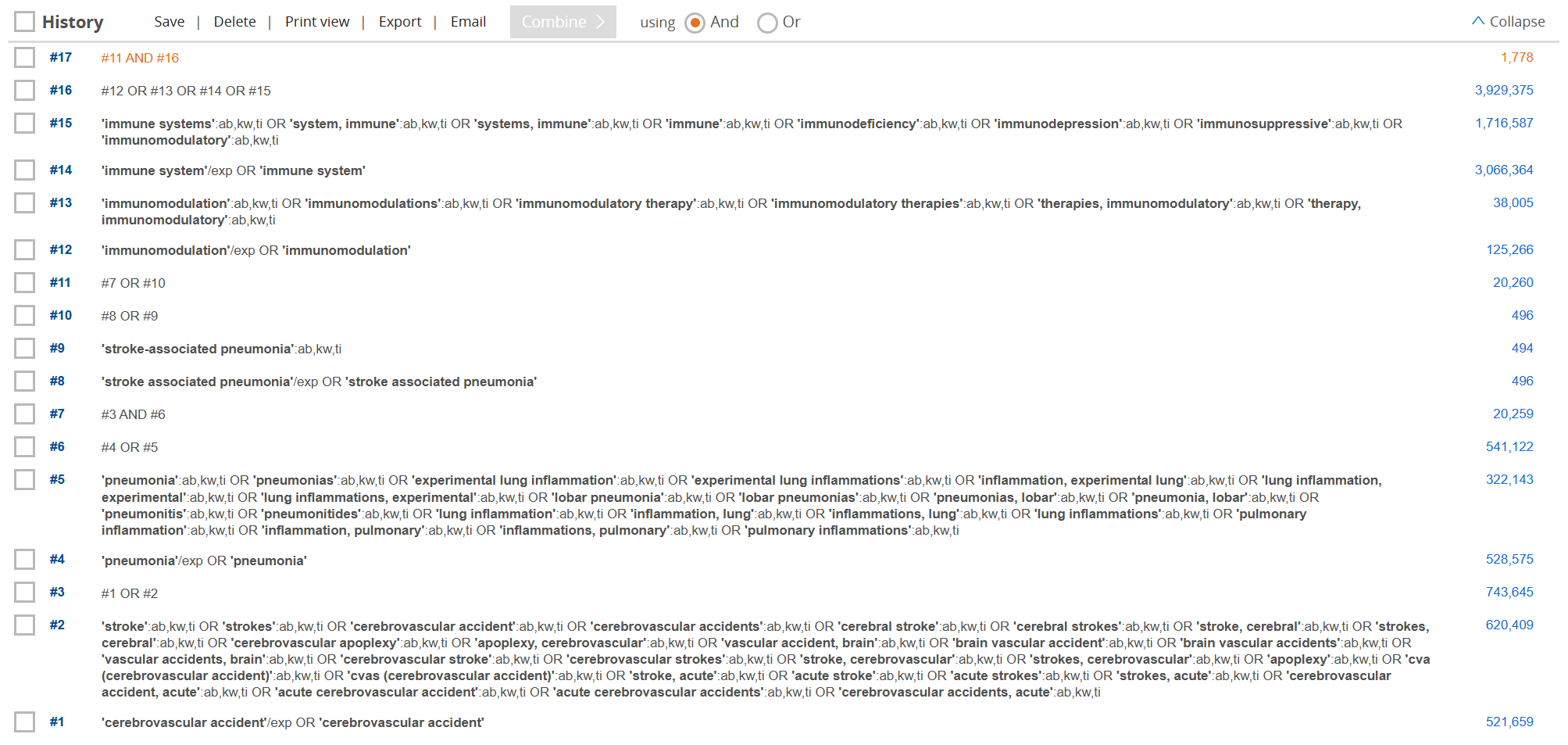

Supplement: Supplementary file 1 [file Table1.doc]
